# Molecular Basis for Lytic Bacteriophage Resistance in Enterococci

**DOI:** 10.1128/mBio.01304-16

**Published:** 2016-08-30

**Authors:** Breck A. Duerkop, Wenwen Huo, Pooja Bhardwaj, Kelli L. Palmer, Lora V. Hooper

**Affiliations:** a Department of Immunology, University of Texas Southwestern Medical Center, Dallas, Texas, USA; b Howard Hughes Medical Institute, University of Texas Southwestern Medical Center, Dallas, Texas, USA; c Department of Biological Sciences, University of Texas at Dallas, Richardson, Texas, USA

## Abstract

The human intestine harbors diverse communities of bacteria and bacteriophages. Given the specificity of phages for their bacterial hosts, there is growing interest in using phage therapies to combat the rising incidence of multidrug-resistant bacterial infections. A significant barrier to such therapies is the rapid development of phage-resistant bacteria, highlighting the need to understand how bacteria acquire phage resistance *in vivo*. Here we identify novel lytic phages in municipal raw sewage that kill *Enterococcus faecalis*, a Gram-positive opportunistic pathogen that resides in the human intestine. We show that phage infection of *E. faecalis* requires a predicted integral membrane protein that we have named PIP_EF_ (for phage infection protein from *E. faecalis*). We find that PIP_EF_ is conserved in *E. faecalis* and harbors a 160-amino-acid hypervariable region that determines phage tropism for distinct enterococcal strains. Finally, we use a gnotobiotic mouse model of *in vivo* phage predation to show that the sewage phages temporarily reduce *E. faecalis* colonization of the intestine but that *E. faecalis* acquires phage resistance through mutations in PIP_EF_. Our findings define the molecular basis for an evolutionary arms race between *E. faecalis* and the lytic phages that prey on them. They also suggest approaches for engineering *E. faecalis* phages that have altered host specificity and that can subvert phage resistance in the host bacteria.

## IMPORTANCE

Bacteriophage therapy has received renewed attention as a potential solution to the rise in antibiotic-resistant bacterial infections. However, bacteria can acquire phage resistance, posing a major barrier to phage therapy. To overcome this problem, it is necessary to understand phage resistance mechanisms in bacteria. We have unraveled one such resistance mechanism in *Enterococcus faecalis*, a Gram-positive natural resident of the human intestine that has acquired antibiotic resistance and can cause opportunistic infections. We have identified a cell wall protein hypervariable region that specifies phage tropism in *E. faecalis*. Using a gnotobiotic mouse model of *in vivo* phage predation, we show that *E. faecalis* acquires phage resistance through mutations in this cell wall protein. Our findings define the molecular basis for lytic phage resistance in *E. faecalis*. They also suggest opportunities for engineering *E. faecalis* phages that circumvent the problem of bacterial phage resistance.

## INTRODUCTION

*Enterococcus faecalis* is a Gram-positive bacterium that is a natural resident of the mammalian gastrointestinal tract (1). In addition to living a commensal lifestyle, *E. faecalis* is an opportunistic pathogen that causes intestinal dysbiosis and bloodstream infections (2, 3). The enterococci, including *E. faecalis*, have emerged as prevalent hospital-acquired pathogens and have increasingly acquired pathogenic and antibiotic resistance traits (4, 5). Antibiotic resistance in *E. faecalis* is especially troubling considering the emergence of organisms that are resistant to last resort antibiotics such as vancomycin and daptomycin (6–8). In addition, *E. faecalis* can be a conduit for the horizontal transfer of DNA between other opportunistic pathogens such as *Clostridium difficile* and *Staphylococcus aureus* (9, 10). Therefore, new therapeutic strategies are needed to control enterococcal populations both in native intestinal habitats and during hospital outbreaks.

Bacteriophages (phages), viruses that infect and kill bacteria, have long held promise as potential therapeutics (11). With the critical need for novel therapeutics in the fight against multidrug-resistant bacteria, phages are receiving renewed interest for their use as bactericidal agents. Phage therapy has several advantages over broad-spectrum antibiotics. First, phages are highly specific and can be tailored to target a narrow spectrum of bacteria. Second, phage replication is restricted to the environment where the host bacterium resides, and thus phages are self-resolving upon exhaustion of their host reservoir (12). Third, obligate lytic phages cannot integrate into the host bacterial genome as prophages, which limits the chance of introducing phage-carried virulence factors and antibiotic resistance genes into the bacterial genome.

A number of phages infect *E. faecalis* and *Enterococcus faecium* and include both obligate lytic phages and prophages (13). The *E. faecalis* genome harbors multiple prophages that have been implicated in virulence, interspecies competition, and biofilm dispersal (14–16). There are also numerous obligate lytic phages that infect *E. faecalis* and rapidly kill their bacterial hosts (13). These phages show a remarkable degree of specificity for certain *E. faecalis* strains. This specificity suggests that there is an evolutionary “arms race” between *E. faecalis* and its lytic phages, with underlying mechanisms that promote the evolution of phage resistance in the targeted strains and a corresponding ability of the phage to evolve new host strain specificities.

Despite the therapeutic promise of *E. faecalis* phages (17, 18), little is known about *E. faecalis* phage receptors, the molecular basis for phage strain specificity, or how *E. faecalis* develops phage resistance. Phage resistance is an especially formidable barrier for phage therapy, and thus, it is imperative to understand resistance mechanisms in order to develop phage therapies that sidestep this problem. Here we have isolated novel lytic phages from municipal raw sewage that infect *E. faecalis*. We have used these phages to identify an *E. faecalis* membrane protein named PIP_EF_ (for phage infection protein from *E. faecalis*) that promotes lytic phage infection. We find that a variable region in PIP_EF_ specifies phage tropism for distinct *E. faecalis* strains and that mutations in this variable region confer *E. faecalis* phage resistance. We also find that the PIP_EF_ variable region in enterococci from raw sewage is diversified, suggesting that phage-bacterium interactions drive the accumulation of PIP_EF_ variation in environmental *E. faecalis*. Last, we use a gnotobiotic mouse model of phage predation to show that *E. faecalis* acquires phage resistance *in vivo* through mutations in PIP_EF_. Our findings define the molecular basis for an evolutionary arms race between *E. faecalis* and its lytic phages that leads to *E. faecalis* phage resistance.

## RESULTS

### Genome sequence analysis of novel lytic *E. faecalis* bacteriophages.

**FIG 1:**
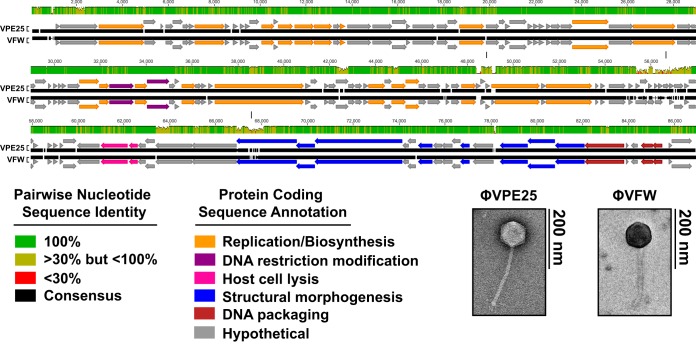
Genome organization of lytic phages φVPE25 and φVFW. Whole-genome alignments were performed using MAFTT version 1.3 (61). Open reading frames for φVPE25 and φVFW were determined using RAST version 2.0, and the resulting data were imported into Geneious 6.0.6. Modular gene organization based on predicted function is color coded. Vertical lines indicate regions with a high degree of nucleotide heterogeneity between φVPE25 and φVFW. Transmission electron microscopy revealed that φVPE25 and φVFW are noncontractile tailed siphophages.

Municipal raw sewage was screened for phages that formed plaques on *E. faecalis* V583. Two phages were isolated and clonally purified by successive agar overlays. These phages formed clear plaques on *E. faecalis* V583. Transmission electron microscopy revealed that the phage morphologies were consistent with the *Siphoviridae* family of noncontractile tailed phages (Fig. 1) (19). We designated these phages φVPE25 and φVFW, where V stands for *E. faecalis* strain V583, on which these phages were isolated, followed by the source of raw sewage (PE25 for primary effluent pump 25 and FW for flocculated water).

The φVPE25 and φVFW genomes are double-stranded DNA consisting of 86,524 bp and 85,865 bp, respectively. Each genome assembled into one large contig with multiple ambiguous nucleotide assignments and low read mapping coverage at the 5′ and 3′ ends suggesting no clear edges at the ends of the contigs. This was consistent with a circularly permuted genome that is terminally redundant. The two genomes are highly congruent, sharing ~95% nucleotide identity, suggesting that these phages recently diverged. A comparative analysis was performed to determine the degree of φVPE25 and φVFW genomic DNA similarity to seven recently characterized siphophages that infect *E. faecalis* (see Fig. S1A in the supplemental material) (20–23). φVPE25 and φVFW have short regions of similarity to these phages but are largely dissimilar at the nucleotide level. The genomes of φVPE25 and φVFW were then compared to the NCBI nonredundant nucleotide database using BLASTn (24). This confirmed that φVPE25 and φVFW have short regions of nucleotide identity to enterococcal phages AUEF3, IME-EF3, EfaCPT1, and EFAP1 and also revealed regions of similarity to *Lactococcus lactis* phage KSY1 and *Bacillus* sp. plasmid pBUYP1 (Fig. S1B) (25). These comparative analyses show that although some nucleotide similarity is observed between φVPE25 and φVFW and other enterococcal siphophages, they are mostly composed of previously unidentified DNA sequence.

Open reading frames (ORFs) were identified and annotated by rapid annotation using subsystem technology (RAST) and BLASTp (24, 26). Approximately 130 ORFs were predicted for both phage genomes. Thirty-seven percent of the ORFs were either assigned a function or were related to other predicted phage genes. The remaining 63% of the ORFs were categorized as hypothetical (see Table S1 in the supplemental material). Similar to other siphophages, the genomes of φVPE25 and φVFW are modular, consisting of genes organized by predicted function (27). These include genes involved in nucleotide biosynthesis and modification, phage particle morphogenesis and DNA packaging, and host cell lysis (Fig. 1 and Table S1). Analysis of these phage gene clusters revealed the presence of a putative recombinase with homology to phage integrase protein family members (Pfam, PF00589). However, integrated phage genomes were not detected in the genomic DNA of *E. faecalis* V583 that had evolved resistance to φVPE25 and φVFW as determined by Southern blotting (Fig. S2). This suggests that φVPE25 and φVFW are obligate lytic phages that are incapable of lysogeny. However, it is possible that these phages emerged from a common prophage ancestor.

### The φVPE25 and φVFW genomes are modified to avoid restriction digestion.

Phages frequently deploy strategies that limit restriction digestion of their genomes by host bacterial endonucleases. This includes chemical modification of specific genomic sequences. Analysis of the φVPE25 and φVFW genomes revealed two ORFs predicted to encode phage DNA-modifying proteins. Both ORFs encode proteins resembling nucleotide sugar synthetase-like enzymes, including a β-glucosyltransferase.

**FIG 2:**
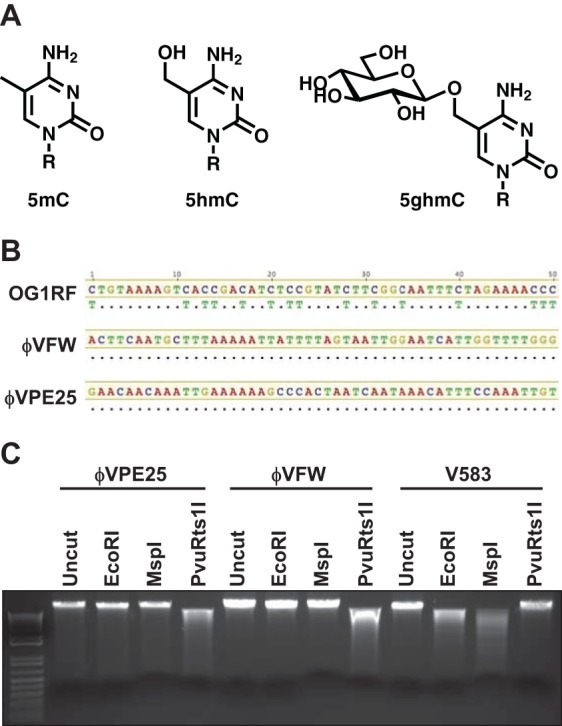
φVPE25 and φVFW DNA is modified at cytosine residues. (A) Structure of methylation and glycosylation modifications that occur at cytosine residues in DNA. Cytosine can be methylated in the form of a single methyl group (5mC) or hydroxyl-methylated (5hmC). 5hmC can be converted to a glucose-linked cytosine (5ghmC) by glucosyltransferase. (B) DNA sequencing analysis of sodium bisulfite-treated *E. faecalis* OG1RF genomic DNA or φVPE25 and φVFW genomic DNA. Unmodified cytosine in *E. faecalis* genomic DNA is converted to uracil after bisulfite treatment and when sequenced appears as thymidine. φVPE25 and φVFW genomic DNA resists bisulfite conversion, confirming cytosine modification. Dots indicate that these nucleotides match those in the consensus sequence. (C) Restriction endonuclease digestion of genomic DNA from φVPE25, φVFW, and *E. faecalis* V583. Incomplete digestion by PvuRts1I may be due to a minimum number of glycosylation sites or to inefficient DNA cleavage.

Phage-encoded β-glucosyltransferases modify hydroxymethylated DNA (28). Cytosine residues are first converted to 5-methylcytosine (5mC) followed by hydroxylation of the methyl group to 5-hydroxymethylcytosine (5hmC). β-Glucosyltransferase then adds a glucose moiety from uridine diphosphoglucose to 5hmC, creating 5-glucose-hydroxymethylcytosine (5ghmC) (Fig. 2A) (29–31). In *Escherichia coli* T-even phages, β-glucosyltransferase activity protects cytoplasmic phage DNA from destruction by restriction endonucleases (32).

To determine whether φVPE25 and φVFW chromosomal DNA is modified, we treated phage and *E. faecalis* genomic DNA with sodium bisulfite which deaminates cytosine, converting it to uracil. However, modified cytosines are protected from conversion (33). After sodium bisulfite treatment, the converted cytosine residues appear as thymine in Sanger sequencing reactions. Both φVPE25 and φVFW genomic DNAs were protected from sodium bisulfite conversion, suggesting that the phage DNAs are modified at cytosine residues (Fig. 2B). Both φVPE25 and φVFW DNAs were resistant to digestion with the restriction enzyme EcoRI, further suggesting that the phage DNAs are modified (Fig. 2C).

To determine whether the DNA modification was methylation or glycosylation, we performed restriction digestions using enzymes that recognize methylated and glycosylated DNA. MspI cleaves unmethylated DNA or DNA containing 5mC and 5hmC but not 5ghmC and was unable to digest the phage DNAs, consistent with the presence of glycosylated cytosine (Fig. 2C). Conversely, PvuRts1I, which can cleave 5ghmC-containing DNA (34), digested both φVPE25 and φVFW DNAs but not the *E. faecalis* genomic DNA control (Fig. 2C). These data suggest that the phage DNAs are modified by glycosylation, likely in the form of glucose moieties.

### The protein EF0858 is essential for phage infection of *E. faecalis.*

We next sought to identify *E. faecalis* genes involved in lytic phage infection. We added φVPE25 and φVFW to *E. faecalis* V583 and assessed infection using a confluent lysis agar overlay assay. The emergence of colonies within zones of lysis suggested that these bacteria were phage resistant. This was confirmed by cross streaking pure cultures of the *E. faecalis* isolates against both φVPE25 and φVFW (see Table S2 in the supplemental material). To determine the nature of the phage resistance, we sequenced the genomes of nine isolates using Illumina HiSeq. The sequencing reads of these nine isolates were mapped to the *E. faecalis* V583 reference genome to identify potential polymorphisms. The mutations in each of the nine isolates mapped to the open reading frame *EF0858*.

*EF0858* encodes an 888-amino-acid protein that is a predicted transmembrane protein in *E. faecalis* V583. *EF0858* is 68% identical and 81% similar to the *Lactococcus lactis* subsp. *lactis* Il1403 and *L. lactis* subsp. *cremoris* MG1363 phage infection protein (PIP), and the N terminus is distantly related (21% identity, 42% similarity) to the *Bacillus subtilis* 168 protein YueB, both of which are integral membrane proteins involved in phage adsorption and infection (35–37). Due to the high similarity of EF0858 to the *L. lactis* phage infection protein, we will refer to EF0858 as PIP_EF_ for phage infection protein from *E. faecalis*. Various mutation types were observed in PIP_EF_, including deletion or insertion polymorphisms (DIPs), single nucleotide polymorphisms (SNPs), and an IS*256* insertion element positioned in the 5′ region of the PIP_EF_ coding sequence (see Table S2 in the supplemental material) (38). In the case of the DIPs and SNPs, each of these mutations produced a frameshift or a nonsense mutation in the form of a premature stop codon (Table S2).

**FIG 3:**
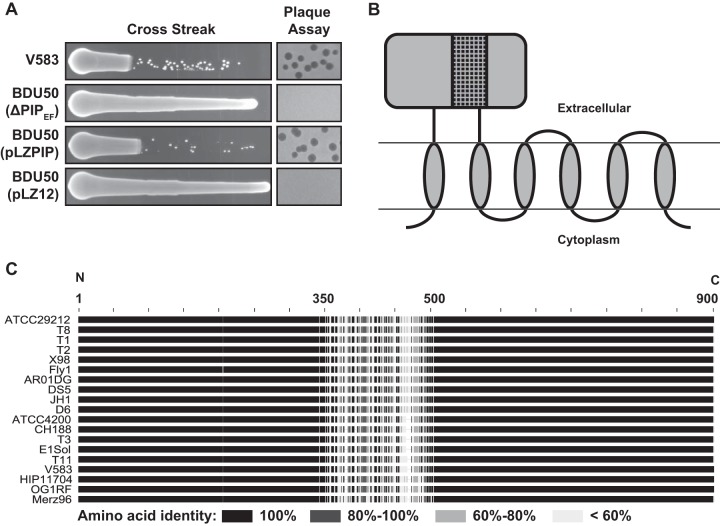
EF0858 encodes PIP_EF_, promotes phage infection, and harbors a hypervariable region. (A) Cross streak and plaque assays using φVPE25 show the susceptibility or resistance profiles of *E. faecalis* V583 and the isogenic PIP_EF_ deletion strain BDU50. Introduction of the pLZPIP plasmid, which contains the entire open reading frame of PIP_EF_ restores phage infectivity of *E. faecalis* BDU50. pLZ12 is the empty vector. (B) Topological cartoon of *E. faecalis* V583 PIP_EF_ generated using TOPCONS (62). The cartoon depicts PIP_EF_ as an integral membrane protein that spans the membrane six times. The ~160-amino-acid variable region of PIP_EF_ is represented as the black box with a black netting pattern in the large extracellular domain. (C) Pairwise amino acid sequence alignments of 19 PIP_EF_ homologs were performed using Geneious 6.0.6. The N and C termini are indicated.

To confirm that PIP_EF_ alone is responsible for the phage resistance phenotype, we constructed an in-frame deletion of PIP_EF_ on the *E. faecalis* V583 chromosome using allelic replacement. Like the phage-resistant isolates in Table S2, the in-frame PIP_EF_ deletion mutant BDU50 was resistant to phage infection as measured by both cross streak and plaque assays (Fig. 3A). Importantly, phage sensitivity could be restored by adding PIP_EF_ on a multicopy plasmid to strain BDU50. Thus, PIP_EF_ is sufficient to promote phage infection in *E. faecalis* V583.

### A PIP_EF_ variable region determines lytic phage tropism.

**FIG 4:**
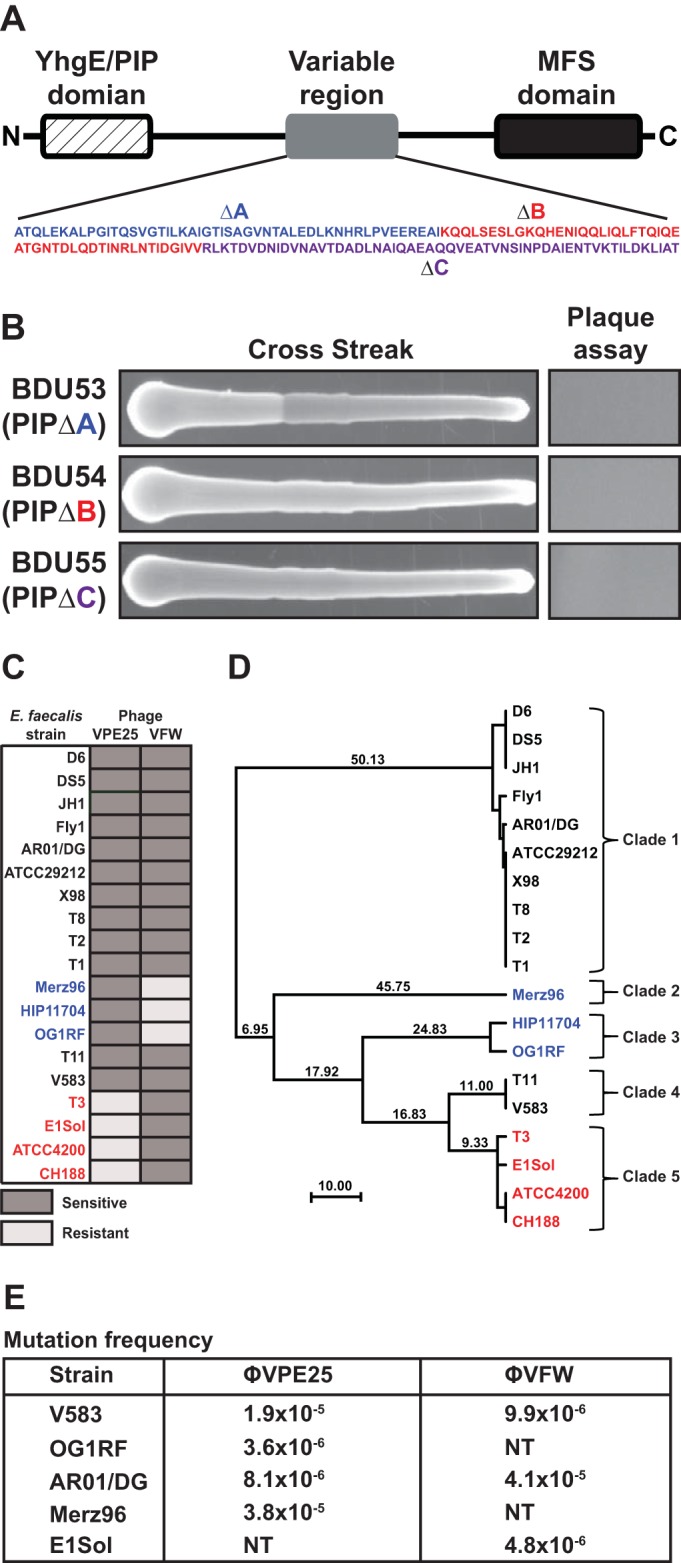
A variable region in PIP_EF_ determines phage tropism in *E. faecalis*. (A) Schematic of PIP_EF_ from *E. faecalis* V583. A variable region covering ~160 amino acids is located in the center of the PIP_EF_ coding sequence. (B) Both φVPE25 cross streak and plaque assays show that truncations in the PIP_EF_ variable region abolish phage infectivity regardless of location. The deletions (ΔA, ΔB, and ΔC) correspond to the colored amino acids highlighted in the magnified area of the variable region shown in panel A. (C) Susceptibility profiles of 19 *E. faecalis* strains for phages φVPE25 and φVFW. (D) Clustering of PIP_EF_ variable region amino acid alignments from 19 strains of *E. faecalis*. Strains cluster according to their susceptibility patterns as determined in panel C. These strains are indicated by color coding as follows: strains sensitive to killing by both phages (black), strains sensitive to only φVPE25 (blue), and strains sensitive only to φVFW (red). Strains can be further grouped into five specific clades based on PIP_EF_ variable region amino acid identity. (E) Representative clade-specific mutation frequencies for phages φVPE25 and φVFW. NT, not tested (due to natural resistance to the phage of interest).

A homolog of *E. faecalis* V583 PIP_EF_ is present in multiple *E. faecalis* strains whose genomes have been sequenced. Amino acid alignment of PIP_EF_ proteins from 19 sequenced *E. faecalis* strains showed a high degree of amino acid identity at the N and C termini, whereas the central region of PIP_EF_, which encompasses approximately 160 amino acids, is highly variable (Fig. 3C). This variable region covers amino acids 342 to 494 in *E*. *faecalis* V583 PIP_EF_ and is part of a large predicted extracellular domain positioned between the first and second transmembrane domains (Fig. 3B and 4A). On the basis of the positioning of the variable region in an extracellular facing domain of PIP_EF_, we hypothesized that this region might play a biological role during phage infection. Indeed, truncations within the variable region of PIP_EF_ rendered *E. faecalis* V583 resistant to infection by φVPE25 (Fig. 4B).

We next queried the spectrum of φVPE25 and φVFW bactericidal activity against 19 *E. faecalis* strains using a cross streak assay. Unique patterns of host tropism emerged for both φVPE25 and φVFW. Twelve of the *E. faecalis* strains were sensitive to killing by both φVPE25 and φVFW, three strains (Merz96, HIP11704, and OG1RF) were killed by only φVPE25 and not φVFW, and four strains (T3, E1Sol, ATCC 4200, and CH188) were killed by φVFW but resisted killing by φVPE25 (Fig. 4C). Interestingly, we noted a relationship between the amino acid relatedness of the PIP_EF_ variable region and the phage infectivity profile. PIP_EF_ variable region sequences clustered into three distinct groups according to phage susceptibility patterns that included strains that were either exclusively killed by φVPE25, exclusively killed by φVFW, or those that were killed by both phages (Fig. 4D). These data suggested that the PIP_EF_ variable region is a phage specificity determinant.

The PIP_EF_ variable region amino acid sequence serves as a predictor of phage susceptibility and allows *E. faecalis* strains to be grouped into clades based on these sensitivity patterns. We propose the following typing scheme based on the PIP_EF_ variable region amino acid sequence for predicting phage sensitivity in *E. faecalis* strains: clade 1, strains D6, JH1, DS5, AR01/DG, T8, ATCC 29212, T2, T1, X98, and Fly1; clade 2, strain Merz96; clade 3, strains OG1RF and HIP11704; clade 4, strains V583 and T11; clade 5, strains T3, CH188, ATCC 4200, and E1Sol. The PIP_EF_ variable region sequence identity cutoff for clade grouping was 95% at the nucleic acid level. The frequency for resistance to φVPE25 and φVFW was determined using a representative *E. faecalis* strain from each phage sensitivity clade (Fig. 4E). All five phage sensitivity clades showed resistance frequencies of 10^−5^ to 10^−6^, and for φVPE25, it appears that clades 2 and 3 are less prone to develop resistance than clades 1 and 4. These data show that spontaneous phage resistance in *E. faecalis* arises at a frequency similar to that of other bacteria (39–41).

### PIP_EF_ swapping alters bacteriophage tropism.

**FIG 5:**
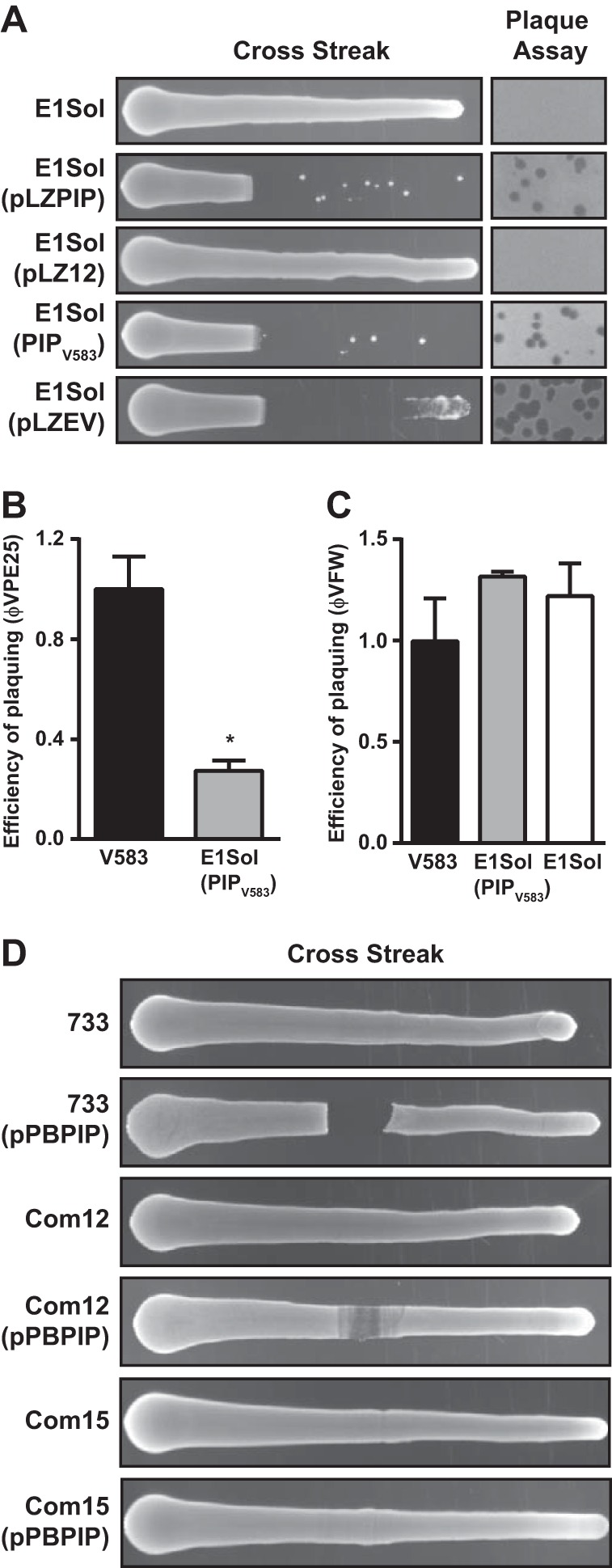
PIP_EF_ swapping alters phage tropism. (A) Using the *E. faecalis* strain E1Sol, which is naturally resistant to infection by φVPE25, both cross streak and plaque assays showed that E1Sol can acquire φVPE25 susceptibility by expressing the *E. faecalis* V583 PIP_EF_ gene (pLZPIP). Single-copy replacement of the *E. faecalis* E1Sol PIP_EF_ homolog with V583 PIP_EF_ in the E1Sol chromosome also confers φVPE25 sensitivity (PIP_V583_). A chimera of *E. faecalis* E1Sol PIP_EF_ and the variable region of V583 PIP_EF_ show that the PIP_EF_ variable region determines phage tropism (pLZEV). (B and C) Plaquing efficiency of φVPE25 (B) and φVFW (C) on *E. faecalis* V583, E1Sol, and PIP_V583_ transgenic E1Sol strains. The value that was significantly different (*P* < 0.01) by Student’s *t* test from the value for *E. faecalis* V583 is indicated by an asterisk. (D) Cross streak assay showing that expression of *E. faecalis* V583 PIP_EF_ from plasmid pPBPIP can confer φVPE25 sensitivity on *E. faecium* strains 1,141,733 and Com12, but not strain Com15.

To further test the idea that PIP_EF_ is an *E. faecalis* phage tropism determinant, we introduced the pLZPIP plasmid, which carries PIP_EF_ from *E. faecalis* V583 (clade 4), into *E. faecalis* E1Sol (clade 5). Strain E1Sol is resistant to infection by φVPE25 (Fig. 4C). However, when carrying pLZPIP, the phage tropism of E1Sol was altered, rendering the strain susceptible to infection by φVPE25 (Fig. 5A). This was confirmed by replacing in-frame the entire portion of the E1Sol PIP_EF_ coding sequence with the V583 PIP_EF_ (PIP_V583_) coding sequence using allelic exchange (Fig. 5A).

To determine whether the PIP_EF_ variable region specified the altered phage tropism of the transgenic E1Sol strain, we constructed the pLZEV plasmid, which contains the entire coding sequence of E1Sol PIP_EF_ except that the variable region is replaced in frame with the V583 PIP_EF_ variable region. Upon introduction of pLZEV into *E. faecalis* E1Sol, phage tropism was altered, resulting in the sensitivity of E1Sol to φVPE25 infection (Fig. 5A). These data show that the PIP_EF_ variable region determines phage specificity for the bacterial host strain. However, we observed that φVPE25 was less efficient at plaquing on the transgenic *E. faecalis* E1Sol-PIP_V583_ strain than it was on its preferred host *E. faecalis* V583, whereas φVFW’s ability to form plaques on E1Sol-PIP_V583_ was unaltered (Fig. 5B and C). This suggests that other factors, such as differences in cell wall composition, help to determine maximal phage infectivity.

We next asked whether PIP_EF_ could direct phage tropism toward a species other than *E. faecalis*. We chose the related species *E. faecium*, focusing on three strains, Com12, Com15, and 1,141,733, that each encode a PIP homolog that is ~65% identical to V583 PIP_EF_. All three strains are resistant to infection by φVPE25 (Fig. 5D). To test whether the tropism of φVPE25 could be redirected by introducing *E. faecalis* V583 PIP_EF_ into these strains, we created the pPBPIP plasmid, which contains the entire coding sequence of V583 PIP_EF_ controlled by its native promoter. Expression of *E. faecalis* V583 PIP_EF_ in *E. faecium* Com12 and 1,141,733 conferred sensitivity to φVPE25, albeit to a lesser extent than that observed when V583 PIP_EF_ was expressed in *E. faecalis* E1Sol. This finding was reinforced by the observation of a zone of clearance in the high phage titer region of cross streak plates for strains Com12 and 1,141,733 (Fig. 5D). Expression of V583 PIP_EF_ in *E. faecium* Com15 did not render this strain sensitive to φVPE25 infection (Fig. 5D). These data show that PIP swapping in the related species *E. faecium* can alter the tropism of lytic enterococcal phages.

Ectopic expression of *E. faecalis* V583 PIP_EF_ in *E. faecium* altered φVPE25 tropism, yet we did not observe visible plaques during the infection when performing an agar overlay experiment. Using *E. faecium* 1,141,733 carrying pPBPIP, we confirmed that the addition of φVPE25 to logarithmically growing bacterial cells retarded growth, suggesting that these phages successfully infect the bacteria (see Fig. S3A in the supplemental material). However, after 2 h of growth in the presence of φVPE25, an accumulation of φVPE25 particles in the culture fluid was not observed (Fig. S3B). We detected phage transcripts after infection in both wild-type and transgenic PIP_EF_ *E. faecium* 1,141,733, suggesting that replicated phage particles were trapped within the bacteria due to inefficient host cell lysis (Fig. S3C). To test for trapped viable phage particles, we treated the cells with lysozyme and sonication. Indeed, both *E. faecium* strains released viable phage particles upon sonication, whereas the control strain *E. faecalis* E1Sol did not (Fig. S3D). These data show that φVPE25 infects *E. faecium* 1,141,733 and to a greater extent if the bacterium is expressing *E. faecalis* V583 PIP_EF_ from plasmid pPBPIP; however, the phages cannot lyse the *E. faecium* cells.

### PIP_EF_ is dispensable for initial phage attachment and is implicated in DNA entry.

**FIG 6:**
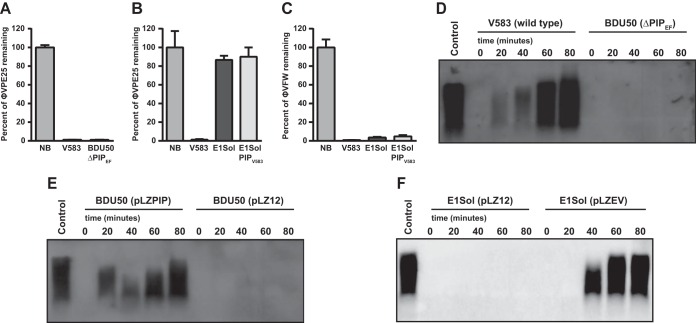
PIP_EF_ promotes DNA entry, but not initial phage adsorption. (A to C) Initial phage adsorption to *E. faecalis* cells was measured by determining the percentage of phages remaining in the supernatant after the addition of various *E. faecalis* strains. NB, no bacteria added. (D to F) Southern blotting was performed using an φVPE25 whole-genome probe on DNA isolated from whole cells infected with φVPE25. (D) *E. faecalis* V583 compared to the isogenic PIP_EF_ mutant strain BDU50. (E) φVPE25 replication can be restored in strain BDU50 when PIP_EF_ is provided in *trans*. (F) The variable region of strain V583 is sufficient to allow φVPE25 DNA entry into *E. faecalis*, because φVPE25 DNA can replicate in strain E1Sol only if the strain expresses a PIP_EF_ chimera carrying the V583 PIP_EF_ variable region (pLZEV) in the large extracellular facing domain. pLZ12 is the empty vector control.

To determine the role of PIP_EF_ during phage adsorption, we used whole bacterial cell pulldown assays to test the ability of phages φVPE25 and φVFW to bind to the *E. faecalis* cell surface. φVPE25 bound to wild-type *E. faecalis* V583 and the isogenic PIP_EF_ mutant BDU50 equally well (Fig. 6A). However, φVPE25 did not bind to the surface of *E. faecalis* E1Sol, even when E1Sol had its PIP_EF_ replaced with *E. faecalis* V583 PIP_EF_ on the chromosome (Fig. 6B). This is in contrast to φVFW which adsorbed to E1Sol, transgenic PIP_V583_ E1Sol, and V583 similarly (Fig. 6C). These data suggest that PIP_EF_ is not required for initial phage adsorption to *E. faecalis*.

We next sought to determine whether PIP_EF_ is involved in phage DNA entry into *E. faecalis* cells. We infected *E. faecalis* strains with φVPE25 and monitored intracellular phage replication over time. As expected, φVPE25 replication was observed in *E. faecalis* V583 but not the PIP_EF_ mutant strain BDU50 (Fig. 6D). φVPE25 replication could be restored in strain BDU50 by plasmid-encoded PIP_EF_ (Fig. 6E). However, φVPE25 could replicate in *E. faecalis* E1Sol only when a plasmid-encoded chimeric PIP_EF_ was introduced into the strain (Fig. 6F). This chimeric PIP_EF_ was comprised of the E1Sol PIP_EF_ sequence except for the variable region, which was from V583 PIP_EF_. These data support the conclusion that the PIP_EF_ variable region facilitates phage DNA entry.

### An environmental reservoir harbors *E. faecalis* with PIP_EF_ diversity.

To characterize PIP_EF_ diversity in a natural environment where *E. faecalis* is endemic, we used culture-dependent and -independent techniques to query the PIP_EF_ variable region in enterococcal isolates from raw sewage. DNA was isolated from total raw sewage and from pooled enterococci that were first isolated from raw sewage by growth on selective agar. PIP_EF_ variable-region-specific PCR was performed, and the resulting amplicons were sequenced using Illumina MiSeq. We first mapped the sequencing reads to a representative from each PIP_EF_-specific clade in order to quantify PIP_EF_ diversity (see Table S3 in the supplemental material). Regardless of whether the PIP_EF_ variable region was directly amplified from total raw sewage DNA or from genomic DNA after selective plating of enterococcal isolates, all five PIP_EF_ clades were present; however, the majority of the PIP_EF_-specific reads matched clades 4 and 5, which include PIP from strains V853 and E1Sol, respectively (Table S3). Interestingly, clade 1 was more highly represented in the sequencing reads from pooled enterococci isolated by selective plating than in those generated in the absence of selection, suggesting that growth on selective agar may allow for the propagation of minority community members that otherwise go undetected by PCR in the absence of selection. These data show that raw sewage is a robust habitat for enterococci harboring diverse PIP_EF_ genes representing all five identified phage sensitivity clades.

We next assessed the amino acid conservation of the PIP_EF_ variable region sequences obtained from total raw sewage and raw sewage enterococcal isolates grown from selective plating. We first performed *de novo* assemblies of the sequencing reads for the PIP_EF_ variable region and aligned the resulting contigs to the *E. faecalis* V583 PIP_EF_ variable region as a reference (see Fig. S4A in the supplemental material). The majority of the PIP_EF_ variable region amino acid content from both total raw sewage and raw sewage enterococcal isolates was identical to the amino acid sequence of the *E. faecalis* V583 PIP_EF_ variable region. This is consistent with the observation that most PIP_EF_ variable region reads mapped to strain V583 at the nucleotide level (Table S3). However, assemblies carrying a number of amino acid variants were identified in a region that is divergent at the amino acid level between clade 4 and clade 5 within the PIP_EF_ variable region (Fig. S4B). These residues may be responsible for directing phage specificity within PIP_EF_ and suggest that PIP_EF_ diversity is maintained in the environment.

### Phage-mediated killing of *E. faecalis* drives the emergence of PIP_EF_ mutants *in vivo*.

Enterococcal overgrowth within the human intestinal tract can occur in response to broad-spectrum antibiotics or immunosuppressive drugs (2, 42). This overgrowth can result in intestinal dysbiosis that puts patients at risk of acquiring a secondary septic enterococcal infection. With the rise of multidrug-resistant strains of *E. faecalis* and *E. faecium*, novel therapeutic approaches to combat such infections are needed.

**FIG 7:**
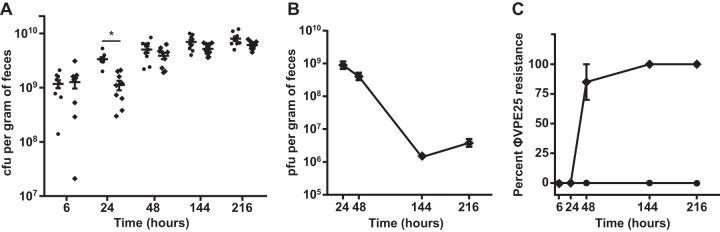
*In vivo* phage predation selects for *E. faecalis* PIP_EF_ mutants. Germfree C57BL6/J mice were orally inoculated with *E. faecalis* V583 followed by an oral treatment of φVPE25. (A) Fecal burden of *E. faecalis* from mice with and without phage treatment over a 9-day period. Each symbol represents the value for an individual mouse, and the short black bar represents the mean of the group of mice. The means that are significantly different (*P* < 0.001) by Student’s *t* test are indicated by a bar and asterisk. (B) φVPE25 particle numbers from gnotobiotic mouse feces as determined by a plaque assay. (C) Percentage of φVPE25-resistant *E. faecalis* clones isolated from gnotobiotic mice following phage treatment. The percentage of φVPE25 resistance was calculated by determining the number of phage-resistant isolates by cross streaking and then dividing the number of resistant isolates by the total number of isolates acquired at each time point and multiplying by 100. Symbols: ♦, treated with φVPE25; ●, not treated with phage.

We sought to test the ability of phage φVPE25 to kill *E. faecalis* growing in the intestinal tract. We established a gnotobiotic mouse model of *E. faecalis* intestinal colonization in which germfree C57BL6/J mice were orally inoculated with *E. faecalis* V583. After a 6-h colonization period, φVPE25 was orally administered to the mice. Starting at 6 h and extending to 216 h (9 days) after φVPE25 administration, feces were collected from each mouse, and viable *E. faecalis* cells were enumerated by colony counting. After 24 h of φVPE25 treatment, there was a threefold drop in total *E. faecalis* intestinal loads compared to non-φVPE25-treated animals. This suggests that φVPE25 can modestly reduce the *E. faecalis* numbers in the mouse intestine (Fig. 7A). However, as early as 48 h and through day 9 of the experiment, the *E. faecalis* community rebounded to the level of colonization observed for untreated animals, even when the phages were added to the drinking water for continuous administration (Fig. 7A). The number of phage particles recovered from feces was the highest at 24 and 48 h after φVPE25 treatment (Fig. 7B). Phage particles were maintained in the intestines of these mice for the duration of the experiment, but the number of recoverable phage particles significantly decreased by 144 h (6 days) and 216 h (9 days) of treatment (Fig. 7B). Thus, the intestinal *E. faecalis* population became φVPE25 resistant.

To test for the acquisition of phage resistance at each time point, we screened a total of 40 *E. faecalis* fecal isolates from gnotobiotic mice that received either an φVPE25 or saline control inoculation. After 6 h of φVPE25 treatment, 100% of isolates remained sensitive to φVPE25 infection as determined by cross streaking (Fig. 7C). By 24 h after φVPE25 treatment, the number of *E. faecalis* isolates that were φVPE25 resistant rose to approximately 85%, and the number of resistant clones increased to 100% by day 9 (Fig. 7C). Thus, *E. faecalis* acquired widespread phage resistance after exposure to lytic phage in the gastrointestinal tract.

We next determined whether these phage-resistant isolates had intact PIP_EF_ ORFs. At 48 h after colonization, a total of 20 *E. faecalis* fecal isolates were collected from both φVPE25-treated and saline-treated mice. Genomic DNA was purified from these isolates and used in PCRs that amplified the PIP_EF_ coding sequence starting175 bp upstream of the predicted translational start site. The integrity of the PIP_EF_ coding sequence was determined using automated Sanger DNA sequencing. PIP_EF_ from all 20 fecal isolates collected from the saline-treated control group had a sequence identical to PIP_EF_ from wild-type *E. faecalis* V583 (data not shown) and remained φVPE25 sensitive (Fig. 7C). However, the 20 fecal isolates from φVPE25-treated mice all had polymorphisms that localized to the PIP_EF_ open reading frame. The polymorphisms that occurred in PIP_EF_ from the fecal isolates included insertion/deletion mutations, frameshift mutations, and insertion sequence mutations (see Table S4 in the supplemental material).

These data show that phage predation on *E. faecalis* in the intestine exerts a strong selective pressure for the development of phage resistance. More importantly, our findings establish that *E. faecalis* acquires phage resistance *in vivo* through mutations in PIP_EF_.

## DISCUSSION

Novel antimicrobial strategies are urgently needed to control increasingly frequent antibiotic-resistant bacterial infections. Phages have long held promise as potential antibacterial therapeutics, and thus, a revival in phage therapy has begun over the last several years. This has led to a renaissance in an understanding of the basic molecular biology of phages and their contributions to host-pathogen interactions (43). This is especially relevant for Gram-positive pathogens, such as *E. faecalis*, that place a heavy burden on the health care system. However, before phages can be routinely used as antienterococcal therapeutics, a detailed understanding of how they interact with their bacterial hosts and the mechanisms that enterococci use to avoid phage infection need to be delineated.

In this study, we have discovered two previously unidentified lytic siphophages, φVPE25 and φVFW, which infect *E. faecalis*. These phages are unique and have little genomic similarity to previously identified siphophages that infect *E. faecalis*. We found that φVPE25 and φVFW harbor genes encoding DNA-modifying enzymes that allow the phages to glycosylate their DNA at cytosine residues as a possible mechanism to avoid host restriction. This is inferred from the presence of a phage-encoded β-glucosyltransferase that is ~40% identical to the enterobacterial phage T4 β-glucosyltransferase, which modifies T4 DNA at 5hmC residues with glucose (31). Our findings are among the first to suggest that Gram-positive phages can modify their DNA by glycosylation (44).

Interestingly, there is a second open reading frame, encoded in the φVPE25 and φVFW genomes, that is annotated as a nucleotide sugar synthetase-like protein. The protein has 28% identity to β-1,6-galactofuranosyltransferase, a member of a class of enzyme that catalyzes the transfer of galactofuranose from UDP-galactofuranose to α-d-glucopyranosides (45). It is possible that the φVPE25/φVFW-encoded enzyme could further modify the glycosylated phage DNA. Genome sequence analysis did not uncover any putative virulence factors or antibiotic resistance genes, suggesting that φVPE25 and φVFW should be considered candidate phages to be further studied and possibly modified for therapeutic applications.

Our studies of φVPE25 and φVFW led us to identify PIP_EF_ as an *E. faecalis* integral membrane protein that is essential for phage infection. We used the phages to select for phage-resistant mutants of *E. faecalis* V583 and found that genomic mutations associated with resistance were clustered in the PIP_EF_-encoding reading frame *EF0858*. PIP_EF_ is orthologous to *L. lactis* PIP, which promotes phage binding and infection (35, 36). PIP_EF_ harbors two distinctive domains: an N-terminal YhgE/PIP domain that is conserved in all *Firmicutes* that harbor a PIP ortholog and lacks a known function and a C-terminal major facilitator superfamily (MFS) domain that might play a role in small-molecule transport.

PIP_EF_ is conserved among *E. faecalis* strains, suggesting that PIP_EF_ performs an important biological function in *E. faecalis*. Interestingly, a second protein containing the YhgE/PIP domain was identified in 8 of the 19 *E. faecalis* strains used in this study. This protein has 21% sequence identity to the N-terminal YhgE/PIP domain of PIP_EF_ and resides in a cluster of genes annotated to function as a type VIIb secretion system (46). This protein is an ortholog of the *S. aureus* EsaA type VIIb secretion system protein and has a similar predicted topology of six transmembrane domains and a C-terminal MFS domain as observed for PIP_EF_ (47). We therefore speculate that PIP_EF_ may be involved in the transport of a small molecule or function in concert with a type VIIb secretion system to secrete effector proteins across the bacterial membrane.

PIP_EF_ harbors a 160-amino-acid region with marked sequence diversity. This protein region is centrally located within the first predicted extracellular domain of PIP_EF_ and can be used to group *E. faecalis* strains based on their phage susceptibility profile. We found that this variable region is both necessary and sufficient to drive phage tropism for specific host bacteria. Raw sewage harbored *E. faecalis* with diverse PIP_EF_ variable regions, suggesting that sewage phages coevolved with diverse *E. faecalis* strains. The changing abundance of such strains due to phage predation may drive the emergence of phages with altered tropisms for PIP_EF_ variants.

Variable regions of cell wall-embedded proteins have been implicated in phage specificity for Gram-negative bacteria (48–50). In Gram-positive bacteria, the study of how variation in bacterium-encoded phage receptors impacts phage tropism has been limited to cell wall-associated polysaccharides (51, 52). To our knowledge, our findings constitute the first description of a diversifying region of a bacterial membrane protein phage receptor in Gram-positive bacteria.

A key remaining question is the identity of the phage antireceptor that interacts with PIP_EF_. Known phage antireceptors are components of the phage tail (53–55). Several phage tail genes are clustered together in the φVPE25 and φVFW genomes. One particular gene, represented by orf_112 in φVPE25 and orf_110 in φVFW, is a candidate for the antireceptor as it is orthologous to *Streptococcus thermophilus* phage antireceptors with hypervariable regions that specify phage tropism (56).

The PIP_EF_ variable region sequences allowed clustering of *E. faecalis* strains into clades based on PIP_EF_ homology and phage sensitivity. With the continued classification of lytic phages that target *E. faecalis* through a PIP_EF_-dependent mechanism and their assignment to the infection of specific clades, we envision the use of PIP_EF_ variable region sequencing as a precursor for the selection of potential therapeutic phages that could be used to selectively kill *E. faecalis* outbreak strains.

Using a gnotobiotic mouse model of *in vivo* phage predation, we found that *E. faecalis* acquires phage resistance through mutations in PIP_EF_. Lytic phages can only modestly reduce *E. faecalis* numbers in the intestines of gnotobiotic mice, and *E. faecalis* numbers rebound due to the selective overgrowth of PIP_EF_ mutants. Thus, prophylactic phage administration provides a strong selective pressure for the emergence of PIP_EF_ mutations in *E. faecalis*.

The rapid development of phage resistance is just one of several barriers to deploying phages therapeutically (57). Although these phages are specific killers of *E. faecalis*, their exquisite selectivity for a target host enables the development of phage infection resistance and highlights a potential barrier to phage monotherapies against *E. faecalis*. We suggest that our identification of a key driver of phage resistance in *E. faecalis* could facilitate the engineering of phages with altered tropism. Likewise, once identified, the phage antireceptor specific for PIP_EF_ could be modified by site-directed mutagenesis in hopes of loosening its specificity for cell wall targets. Chemical mutagenesis could also be performed on the phage genomes to create phages with altered tropisms. These approaches could enable the development of phage cocktails that circumvent the problem of resistance and could thus be used to treat *E. faecalis* infections.

## MATERIALS AND METHODS

### Bacterial strains and bacteriophages.

A complete list of bacterial strains and phages used in this study can be found in Table S5 in the supplemental material. *E. faecalis* and *E. faecium* were grown statically in brain heart infusion (BHI) broth or on BHI agar at 37°C. *Escherichia coli* was grown in Lennox L broth (LB) with shaking or on LB agar at 37°C. Chloramphenicol was added at 15 µg/ml for *E. faecalis* and *E. faecium* or 8 µg/ml for *E. coli* when needed. Enterococci from municipal raw sewage were isolated using Enterococcosel agar (Becton Dickinson). Growth conditions for the generation of mutant strains of *E. faecalis* by allelic exchange were as specified by Thurlow et al. (58). For a more detailed description of bacterial growth conditions and for plasmid construction, see Text S1 and Table S5 in the supplemental material, respectively.

Phages φVPE25 and φVFW were isolated from untreated raw sewage obtained from a Dallas-Fort Worth water reclamation facility in Texas. Fifty milliliters of raw sewage was centrifuged at 3,220 × *g* for 10 min at room temperature (RT) to sediment large particles. The supernatant was decanted and passed through a 0.45-µm filter. One hundred microliters of clarified sewage was mixed with 130 µl of a 1:10 dilution of an overnight (O/N) culture of *E. faecalis* V583 and then added to Todd-Hewitt broth (THB) top agar (0.35% agar) and poured over a 1.5% agar THB plate. Both top agar and base agar were supplemented with 10 mM MgSO_4_. After O/N growth at 37°C, the resulting plaques were recovered using a sterile Pasteur pipette, and phages were eluted from the agar plugs in 500 µl of SM-plus buffer (100 mM NaCl, 50 mM Tris-HCl, 8 mM MgSO_4_, 5 mM CaCl_2_ [pH 7.4]) O/N at 4°C. Phages were replaqued on *E. faecalis* V583 two more times to ensure that the phages were clonal isolates.

High-titer phage stocks were propagated by infecting 300 ml of logarithmically growing *E. faecalis* V583 at a multiplicity of infection of 0.1 in BHI broth containing 10 mM MgSO_4_. Lysis was allowed to proceed for 4 h at 37°C with shaking. The remaining bacterial cells and debris were pelleted at 4,400 × *g* for 10 min at RT. The culture supernatant was filtered through a 0.45-µm membrane and treated with 5 µg/ml each of DNase and RNase at RT for 1 h, and phages were precipitated by adding 1 M NaCl and 10% (wt/vol) polyethylene glycol 8000 (PEG 8000) and incubated on ice O/N at 4°C. Phages were pelleted by centrifugation at 11,270 × *g* and resuspended in 2 ml of SM-plus buffer. One-third volume of chloroform was added with shaking, and the phases were separated by centrifugation at 16,300 × *g*. The aqueous phase containing the phages was subjected to further purification using cesium chloride centrifugation as described previously (59). Phages were enumerated using the THB agar overlay plaque assay described above.

### Phage cross streak assays.

We used cross streaking to determine the sensitivity of various *E. faecalis* and *E. faecium* strains to phages φVPE25 and φVFW. A total of 10^9^ to 10^10^ phage particles were streaked down the center of a THB agar plate containing 10 mM MgSO_4_ with or without 15 µg/ml chloramphenicol when necessary. Ten microliters of an O/N bacterial culture was spread horizontally across the phage streak. The plates were incubated at 37°C O/N, and bacterial strain sensitivity to a particular phage was indicated by limited to no bacterial growth within and beyond the phage streak area.

### Whole-genome sequencing.

The genomes of phages φVPE25 and φVFW and *E. faecalis* phage-resistant isolates were sequenced by Tufts University Core Facility (TUCF) Genomics, Tufts University. The phage genomes were sequenced using Illumina MiSeq paired-end 250-bp DNA sequencing with an average coverage depth of 881× for φVPE25 and 903× for φVFW. The *E. faecalis* phage-resistant isolates were sequenced using Illumina HiSeq2000 single-end 100-bp DNA sequencing. Variant coverage information for these strains can be found in Table S2. Libraries were prepared with the Nextera XT library preparation kit. All assemblies were performed using CLC Workbench (Qiagen). For additional information about DNA sequencing and for explanations of bioinformatic applications, refer to Text S1.

### Animals.

Germfree C57BL6/J mice were reared at University of Texas (UT) Southwestern Medical Center under sterile conditions as previously described (60). Gnotobiotic *E. faecalis*-colonized mice were established by orally inoculating male C57BL6/J mice with 5 × 10^7^ CFU of *E. faecalis* V583. Intestinal colonization levels were determined by homogenizing fresh fecal pellets in 1 ml of sterile phosphate-buffered saline (PBS) and performing colony counting on Enterococcosel agar. When appropriate, φVPE25 was administered by orally gavaging mice with 1 × 10^10^ PFU and by administering phage in drinking water at a concentration of 5 × 10^8^ PFU/ml. All animal protocols were approved by the Institutional Animal Care and Use Committee of UT Southwestern Medical Center.

### Accession numbers.

All sequences generated for this study have been deposited in the European Nucleotide Archive. The following accession numbers have been assigned: PRJEB13004 (φVPE25 assembled genome), PRJEB13155 (φVFW assembled genome), PRJEB13005 (*E. faecalis* phage-resistant isolates), and PRJEB13161 (PIP_EF_ raw sewage amplicons).

## SUPPLEMENTAL MATERIAL

Text S1  Supplemental Materials and Methods. Download Text S1, PDF file, 0.1 MB

Figure S1  Comparative analysis of the φVPE25 and φVFW genomes. (A) Comparative whole-genome alignments of φVPE25 and φVFW and seven other siphophages that infect *E. faecalis* were performed using Mauve 2.3.1 (A. E. Darling, B. Mau, and N. T. Perna, PLoS One **5**:e11147, 2010, http://dx.doi.org/10.1371/journal.pone.0011147). Lines indicate genomic regions that have connectivity based on nucleotide sequence similarity. (B) A BLASTn analysis was performed on φVPE25 and φVFW to identify genome relatedness to phages outside of known enterococcal siphophages. φVPE25 was set as the reference sequence for circular alignment using Brig 0.95 (N. F. Alikhan, N. K. Petty, N. L. Ben Zakour, and S. A. Beatson, BMC Genomics **12**:402, 2011, http://dx.doi.org/10.1186/1471-2164-12-402). Download Figure S1, PDF file, 1.4 MB

Figure S2  Phage-resistant *E. faecalis* isolates do not harbor integrated prophages. Genomic DNA isolated from *E. faecalis* cells that developed spontaneous resistance to φVPE25 and φVFW infection (see Table S2 in the supplemental material) lacks detectable phage DNA as determined by Southern blotting. The NS-mix lane contains a pool of *E. faecalis* V583 phage-resistant isolates recovered from scraping the soft agar of a semiconfluent φVPE25 lysis plate that was serially passaged in BHI three times prior to extraction of total genomic DNA for Southern blot analysis. The V583 lane contains genomic DNA from phage-sensitive wild-type *E. faecalis* V583. Purified φVPE25 and φVFW DNAs are included as controls. Download Figure S2, PDF file, 0.8 MB

Figure S3  φVPE25 infection of transgenic *E. faecium* 1,141,733. (A) Growth kinetics of *E. faecium* 1,141,733 carrying the *E. faecalis* V583 PIP_EF_ expression plasmid pPBPIP in the presence (●) and absence (♦) of φVPE25. The arrow indicates the time of φVPE25 addition to the culture. (B) φVPE25 particle numbers from infected *E. faecalis* V583 cells carrying pPBPIP immediately after phage addition (Input) or 2 h after phage infection (2 hours post). (C) Quantitative real-time PCR of the φVPE25 transcripts orf_106 (lysin), orf_117 (major tail protein), and orf_123 (major capsid protein) isolated from *E. faecium* 1,141,733 or *E. faecium* 1,141,733 carrying plasmid pPBPIP. *E. faecalis* V583 and the PIP_EF_ mutant strain BDU50 are included as controls. Transcript abundances are plotted on a logarithmic scale. (D) Viable phage particles recovered from wild-type and PIP_EF_ transgenic *E. faecium* 1,141,733 after cell disruption using lysozyme and sonication. *E. faecalis* E1Sol was included as a control strain that is resistant to φVPE25 infection. ND, none detected. Download Figure S3, PDF file, 0.1 MB

Figure S4  Sequence variation among the PIP_EF_ variable regions of *E. faecalis* sewage isolates. (A) Schematic of the variable region of PIP_EF_ (amino acids 342 to 494). The amino acids where variation was detected by direct PCR from raw sewage (EBOX) or from pooled enterococcal isolates grown on selective agar (P1) are indicated in red and green, respectively. For both EBOX and P1 samples, the majority of the amino acid content of the PIP_EF_ variable region matched *E. faecalis* V583 (52.00% of contigs for P1 and 41.61% for EBOX). The top four or five representative contigs containing variant amino acid composition compared to the *E. faecalis* V583 PIP_EF_ variable region sequence as a reference are indicated. (B) Alignment of the *E. faecalis* V583 (clade 4) and E1Sol (clade 5) PIP_EF_ variable regions. Download Figure S4, PDF file, 1.4 MB

Table S1  Enterococcal bacteriophage genome organization and features. Table S1, PDF file, 0.2 MB

Table S2  Spontaneous mutations in EF0858 (PIP_EF_) result in phage resistance. Table S2, PDF file, 0.04 MB

Table S3  Sewage PIP read mapping to clade-specific PIP_EF_ variable region. Table S3, PDF file, 0.04 MB

Table S4  Mutations conferring φVPE25 resistance in *E. faecalis* from gnotobiotic mouse feces. Table S4, PDF file, 0.04 MB

Table S5  Bacterial strains, phages, plasmids, and primers used in this study. Table S5, PDF file, 0.1 MB
