# Supplementary material for: Molecular Basis for Lytic Bacteriophage Resistance in Enterococci
Source: mBio. 2016 Aug 30;7(4):e01304-16. doi: 10.1128/mBio.01304-16 (PMC4999554; doi:10.1128/mBio.01304-16)
Supplement: Text S1 — Supplemental Materials and Methods. Download [file mbo004162963s1.pdf]

Supplementary Materials and Methods

DNA purification and recombinant DNA. *E. faecalis* genomic DNA was purified using a Puregene Yeast/Bact. Kit (Qiagen) with 15 mg/ml lysozyme added to the cell suspension buffer and omitting the kit-provided lytic enzyme solution. For the isolation of bacterial DNA from municipal raw sewage, 50 ml of raw sewage was centrifuged at 290*g* for 5 min to sediment large debris. The supernatant was transferred to a new vessel and centrifuged at 3220*g* for 10 min to pellet bacterial cells. The bacterial cell pellet was resuspended in 0.5 ml of TE buffer (10 mM Tris-HCl, 0.1 mM EDTA, pH 8.0) containing 15 mg/ml lysozyme and incubated at 37ºC for 1 hour. The DNA was extracted with an equal volume of phenol/chloroform followed by precipitation with isopropanol.

Phage DNA was purified by incubating phages with 50 µg/ml proteinase K and 0.5% sodium dodecyl sulfate at 56ºC for 1 hour followed by extraction with an equal volume of phenol/chloroform. The aqueous phase was extracted a second time with an equal volume of chloroform and the DNA was precipitated using isopropanol.

All PCR reactions used for cloning and PIP_{EF} gene sequencing were performed with high fidelity KOD Hot Start DNA Polymerase (EMD Millipore). Plasmids used for allelic exchange contained the pLT06 backbone and chromosomal specific deletion products were generated by overlap extension PCR (1, 2). Complementation was performed using plasmids pLZ12 and a derivative of pAT28 carrying the chloramphenicol acetyltransferase gene (3, 4, unpublished Rodrigues and Palmer). DNA Sanger sequencing of PIP_{EF} PCR products generated from clonal *E. faecalis* isolates recovered from gnotobiotic mouse feces was performed by the UT Southwestern Center for Human Genetics.

Bacteriophage infection of PIP_{EF} transgenic *E. faecium*. To monitor the growth kinetics of *E. faecium* 1,141,733 pPBPIP during phage infection, ϕVPE25 was added at an MOI of 10 to logarithmically growing cells in BHI supplemented with 10 mM $MgSO_4$. To determine if viral progeny were propagated and released from bacterial cells upon infection of *E. faecium* 1,141,733 pPBPIP with ϕVPE25, phages were added to logarithmically growing bacteria at an

MOI of 0.1 and after 2 hrs, 250 µl of culture was collected. 1/3 volume of chloroform was added and the cultures were shaken vigorously. The chloroform was separated by centrifugation at 16,300*g* and phages in the aqueous phase were titered using a THB agar overlay plaque assay with *E. faecalis* V583 as the host. To test if ϕVPE25 virions were produced but not released from bacterial cells, bacterial cultures were grown to logarithmic phase, infected with ϕVPE25 (MOI of 10) for 1 hr and 4 ml of cells were collected and pelleted by centrifugation at 3220*g* for 5 min. The bacterial pellet was washed 5 times with an equal volume of phosphate buffered saline (PBS) and treated with 1 mg/ml lysozyme in 4 ml of PBS for 30 min at 37ºC. The cells were removed from the lysozyme by centrifugation, resuspended in an additional 4 ml of PBS and disrupted using a Misonix S-4000 ultrasonic liquid processor with amplitude of 40 and a pulse time of 3 min (pulse on=10 sec, pulse off=30 sec). After sonication the cell debris was pelleted by centrifugation at 3220*g* for 5 min and the supernatant was filtered (0.45 µm). Released phage particles were enumerated using the THB agar overlay plaque assay with *E. faecalis* V583 as the host.

Mutation Frequency. To determine the mutation frequency of *E. faecalis* strains for phages ϕVPE25 and ϕVFW, O/N cultures were subcultured to an OD_{600} of 0.025 in BHI and grown statically for 5 hours at 37ºC. Bacteria were serially diluted and spotted onto THB top agar overlay plates embedded with 10^9 pfu of either ϕVPE25 or ϕVFW or onto standard THB agar plates to quantify total bacterial numbers from each culture. The plates were incubated for 24 hrs at 37ºC and colonies were counted. Mutation frequency was calculated by dividing the number of resistant colonies found on the phage containing top agar plates by the total number of bacteria from each culture without exposure to phage.

Phage adsorption assays. To determine the ability of ϕVPE25 or ϕVFW to adsorb to the surface of *E. faecalis* cells, 10^7 pfu of phages were added to 500 µl of SM-plus buffer followed by the addition of 10^8 cfu of the appropriate *E. faecalis* strain. After incubation at RT for 10 min, the bacteria were pelleted by centrifugation and the supernatant was enumerated for remaining phage particles using a plaque assay.

Monitoring phage DNA replication in bacterial cells. *E. faecalis* strains were grown in 100 ml of BHI to logarithmic phase with shaking at 37ºC after which $MgSO_4$ was added to a final concentration of 10 mM and ϕVPE25 at an MOI of 0.5. 15 ml aliquots of culture were collected at 0, 20, 40 60 and 80 min post phage addition and added to an equal volume of ice cold acetone:ethanol (1:1) to stop DNA replication. The cells were incubated on ice for at least 10 minutes and harvested by centrifugation at 3220*g,* resuspended in 25 ml of sterile PBS and centrifuged. The cell pellets were stored at -20ºC for 1 hr, resuspended in 600 µl of PBS, and total nucleic acid was extracted using phenol chloroform. A total of 2 µg of DNA was digested with PvuRts1I enzyme for 1.5 hrs at RT and DNA fragments were separated on a 0.7% agarose gel and transferred to a nylon membrane by neutral upward capillary transfer O/N (5). Hybridization and detection was performed using the DIG High Prime DNA labeling and Detection Kit II (Sigma). The probe used was ϕVPE25 genomic DNA digested with PvuRts1I and labeled with digoxigenin-dUTP. Southern hybridizations were either exposed to film and developed on a Konica SRX101A tabletop X-ray film processor or imaged using a ChemiDoc Touch Imaging System (Bio-Rad).

Purification of bacterial RNA. *E. faecalis* and *E. faecium* cultures were grown in BHI with or without antibiotics at 37ºC with shaking to mid logarithmic phase. ϕVPE25 was added to each culture at an MOI of 1 for *E. faecalis* V583 and an MOI of 10 for all other strains. Cultures were infected for 30 min after which 2 ml of culture was collected and added to an equal volume of RNAprotect Bacteria Reagent (Qiagen). The cells were incubated at RT for 5 min and centrifuged at 3220*g* for 5 min. Cell pellets were stored at -20ºC O/N. Once thawed the cells were resuspended in 15 mg/ml lysozyme in TE buffer and incubated at RT for 30 min. 700 µl of RLT lysis buffer (Qiagen) was added and the samples were transferred to 2 ml Lysing Matrix B tubes (MP Biomedicals) and bead beat in a Bullet Blender 5 (Next Advance) at a speed setting of 9 for 1 minute followed by a 1 minute incubation on ice. This was repeated 3 times. Samples were centrifuged at 16,300*g* for 2 minutes and the solution was added to 590 µl of 80% ethanol

and the RNA was purified using an RNeasy Mini Kit (Qiagen) according to the manufacturer's instructions.

cDNA synthesis and real time quantitative PCR. A total of 20 µg of purified bacterial RNA was digested with 8 Kunitz units of DNase from an RNase free DNase Set (Qiagen) for 1 hour at 37ºC. The RNA was purified from the DNase using an RNeasy Mini Kit. 1 µg of RNA was mixed with 250 ng of random hexamer primers (ThermoFisher) and incubated at 65ºC for 5 min. After cooling to RT 200 units of M-MLV Reverse Transcriptase (ThermoFisher) was added and the RNA was reverse transcribed to cDNA (25ºC for 10 min, 42ºC for 1 hr).

cDNA was used as template for real time quantitative PCR (qPCR) with SYBR Green SuperMix-UDG (ThermoFisher) and the following cycling conditions, 50ºC for 2 min, 95ºC for 10 min, followed by 40 cycles of denaturation at 95ºC for 15 sec and elongation at 60ºC for 1 min on a ViiA 7 Real-Time PCR System (ThermoFisher). *E. faecalis* and *E. faecium recA* was used to normalize phage transcripts and transcript levels were calculated using the equation $2^{-\Delta\Delta Ct}$. qPCR primers are listed in Table S5.

Additional DNA sequencing methods and bioinformatics. ϕVPE25 and ϕVFW open reading frame prediction and annotation was done using Rapid Annotation using Subsystem Technology (default parameters) and blastp (best hit cutoff 0.001) (6, 7). For polymorphism analysis of *E. faecalis* phage resistant isolates, raw sequencing reads were mapped to the *E. faecalis* V583 reference genome (AE016830) and the V583 specific plasmids pTEF1 (AE016833), pTEF2 (AE016831) and pTEF3 (AE016832) using Geneious 6.0.6 (8). Polymorphisms were determined using the Annotate and Predict Find Variation/SNP function with the settings minimum coverage=5 and minimum variant frequency=0.9.

High throughput DNA sequencing of the PIP_{EF} variable region from PCR amplified waste water samples and enterococcal waste water isolate genomic DNA was performed by MR DNA (Shallowater, TX) using Illumina MiSeq paired-end 300 bp DNA sequencing. Libraries were generated using the Illumina TruSeq DNA library preparation kit. Variant detection from raw sewage derived PIP_{EF} variable region amplicons was performed by mapping sequencing reads

to the V583 PIP_{EF} gene using CLC Workbench (mismatch cost=3, gap opening cost=3, gap extension cost=3, length fraction=0.8 and similarity fraction=0.8). Variants were detected at 35% frequency and minimal coverage=10. The region with a high density of mutations was extracted together with all the reads that mapped and the allele frequency was calculated by dividing the number of reads that match an allele by the total number of reads extracted.

PIP_{EF} variable region amino acid sequence (defined as amino acids 342-494 from V583 PIP_{EF}) comparisons from 19 *E. faecalis* strains was performed in MacVector. ClustalW was used with the parameters Matrix=Gonnet, gap open penalty=10 and gap extend penalty=0.2. A Phylogenetic tree was built using Method=UPGMA and Distance=the absolute number of differences.

For bisulfite sequencing, 2 µg of ϕVPE25 and ϕVFW DNA was bisulfite-converted using the EpiTect Bisulfite kit (Qiagen) per the manufacturer's instructions. Methyl primers were designed using MethPrimer (9) and can be found in Table S5. The bisulfite-converted phage DNA was amplified using the methyl primers in a standard PCR reaction with Taq polymerase. As a control, 2 µg of *E. faecalis* OG1RF genomic DNA was bisulfite-converted and amplified using methyl primers targeting the open reading frame OG1RF_11844. All PCR products were sequenced by Macrogen (Rockville, MD).

Restriction digestion of phage and bacterial DNA for modification analysis. 1 µg of ϕVPE25, ϕVFW and *E. faecalis* V583 genomic DNA was digested with restriction enzymes according to the manufacturer's specifications. EcoRI and MspI were purchased from New England Biolabs. PvuRts1I was purchased from Active Motif. After restriction digestion, DNA fragments were separated on a 0.7% agarose gel and visualized using ethidium bromide.

Negative staining and transmission electron microscopy. Phages were prepared for and visualized by transmission electron microscopy as previously described (10). Briefly, cesium chloride density gradient-purified phages were applied to glow discharged hydrophilic rendered (40 mA current for 90 seconds) 400 mesh carbon coated copper grids (Ted Pella Inc.) for 1 minute followed by submerging in 0.5% uranyl acetate for 1 minute. The grids were imaged

using a JEOL 2200FS FEG transmission electron microscope. Images were taken in low-dose mode (~20 electron/Å2) at 50,000× with the defocus level varying between −1.2 and −2.0 μm. Images were captured using a 2Kx2K Tietz slowscan CCD camera with a 1.69 postcolumn magnification factor.

1. **Thurlow LR, Thomas VC, Hancock LE.** 2009. Capsular polysaccharide production in *Enterococcus faecalis* and contribution of CpsF to capsule serospecificity. J Bacteriol **191:**6203-6210.
2. **Heckman KL, Pease LR.** 2007. Gene splicing and mutagenesis by PCR-driven overlap extension. Nat Protoc **2:**924-932.
3. **Perez-Casal J, Caparon MG, Scott JR.** 1991. Mry, a trans-acting positive regulator of the M protein gene of *Streptococcus pyogenes* with similarity to the receptor proteins of two-component regulatory systems. J Bacteriol **173:**2617-2624.
4. **Trieu-Cuot P, Carlier C, Poyart-Salmeron C, Courvalin P.** 1990. A pair of mobilizable shuttle vectors conferring resistance to spectinomycin for molecular cloning in *Escherichia coli* and in gram-positive bacteria. Nucleic Acids Res **18:**4296.
5. **Green MR, Sambrook J.** 2012. Molecular cloning : a laboratory manual, 4th ed. Cold Spring Harbor Laboratory Press, Cold Spring Harbor, N.Y.
6. **Aziz RK, Bartels D, Best AA, DeJongh M, Disz T, Edwards RA, Formsma K, Gerdes S, Glass EM, Kubal M, Meyer F, Olsen GJ, Olson R, Osterman AL, Overbeek RA, McNeil LK, Paarmann D, Paczian T, Parrello B, Pusch GD, Reich C, Stevens R, Vassieva O, Vonstein V, Wilke A, Zagnitko O.** 2008. The RAST Server: rapid annotations using subsystems technology. BMC Genomics **9:**75.
7. **Altschul SF, Gish W, Miller W, Myers EW, Lipman DJ.** 1990. Basic local alignment search tool. J Mol Biol **215:**403-410.
8. **Kearse M, Moir R, Wilson A, Stones-Havas S, Cheung M, Sturrock S, Buxton S, Cooper A, Markowitz S, Duran C, Thierer T, Ashton B, Meintjes P, Drummond A.**

2012. Geneious Basic: an integrated and extendable desktop software platform for the organization and analysis of sequence data. Bioinformatics **28:**1647-1649.

9. **Li LC, Dahiya R.** 2002. MethPrimer: designing primers for methylation PCRs. Bioinformatics **18:**1427-1431.

10. **Duerkop BA, Clements CV, Rollins D, Rodrigues JL, Hooper LV.** 2012. A composite bacteriophage alters colonization by an intestinal commensal bacterium. Proc Natl Acad Sci U S A **109:**17621-17626.
