# Supplementary figures and images for: Molecular Basis for Lytic Bacteriophage Resistance in Enterococci
Source: mBio. 2016 Aug 30;7(4):e01304-16. doi: 10.1128/mBio.01304-16 (PMC4999554; doi:10.1128/mBio.01304-16)

Figure S1

A

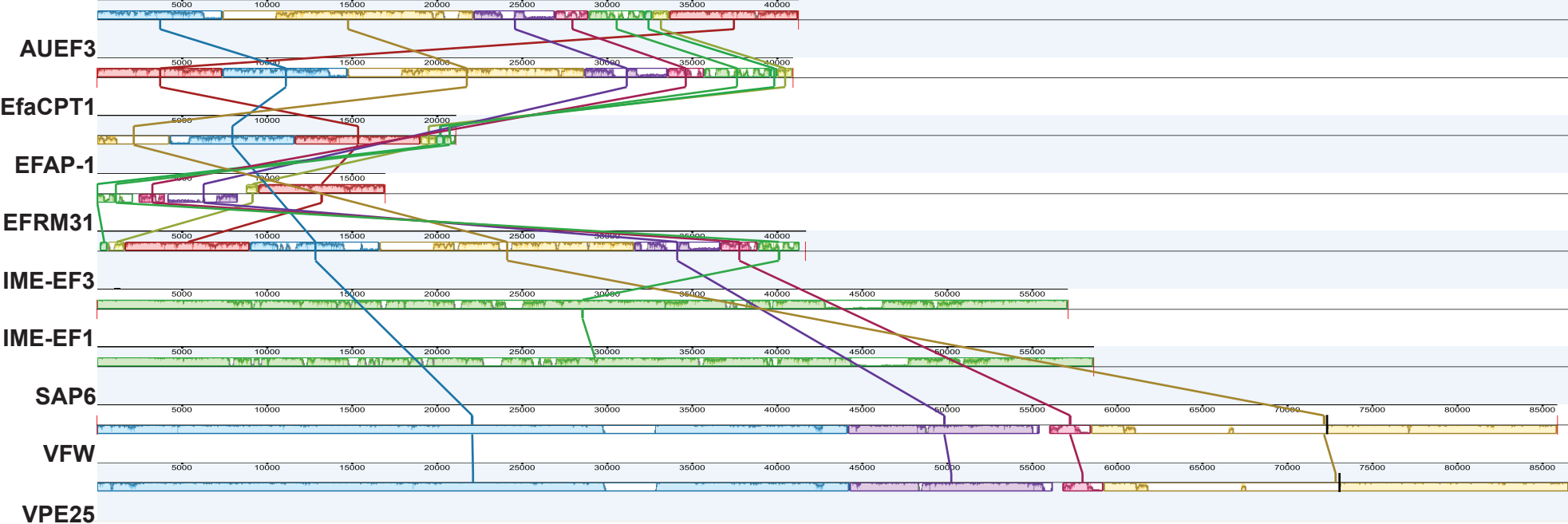

B

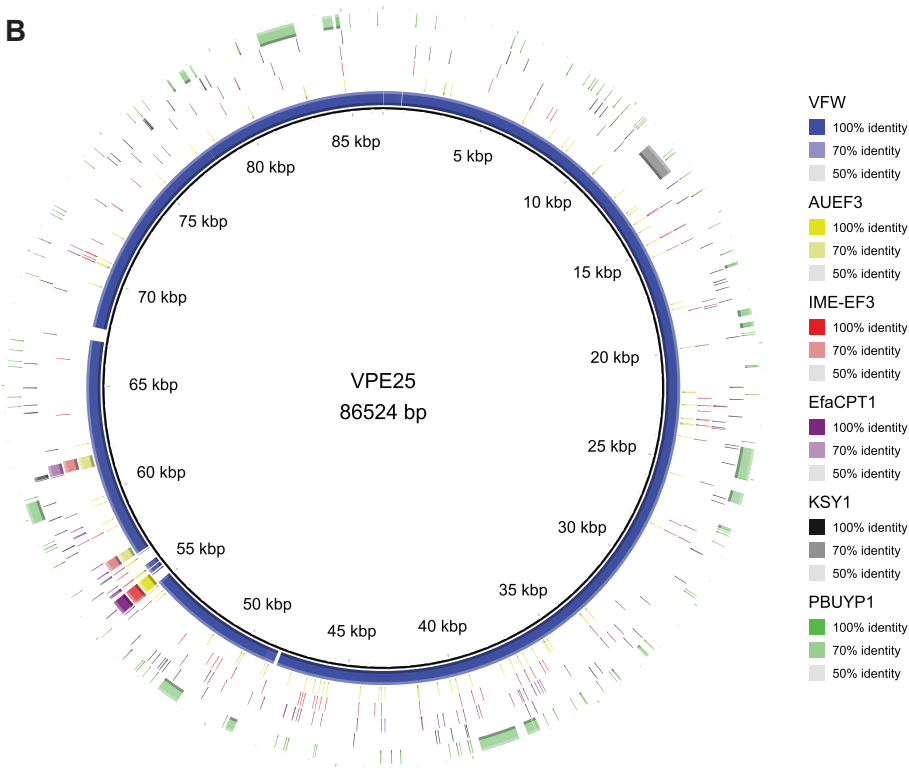

Supplement: Figure S1 — Comparative analysis of the φVPE25 and φVFW genomes. (A) Comparative whole-genome alignments of φVPE25 and φVFW and seven other siphophages that infect E. faecalis were performed using Mauve 2.3.1 (A. E. Darling, B. Mau, and N. T. Perna, PLoS One 5:e11147, 2010, http://dx.doi.org/10.1371/journal.pone.0011147). Lines indicate genomic regions that have connectivity based on nucleotide sequence similarity. (B) A BLASTn analysis was performed on φVPE25 and φVFW to identify genome relatedness to phages outside of known enterococcal siphophages. φVPE25 was set as the reference sequence for circular alignment using Brig 0.95 (N. F. Alikhan, N. K. Petty, N. L. Ben Zakour, and S. A. Beatson, BMC Genomics 12:402, 2011, http://dx.doi.org/10.1186/1471-2164-12-402). Download [file mbo004162963sf1.pdf]

Figure S2

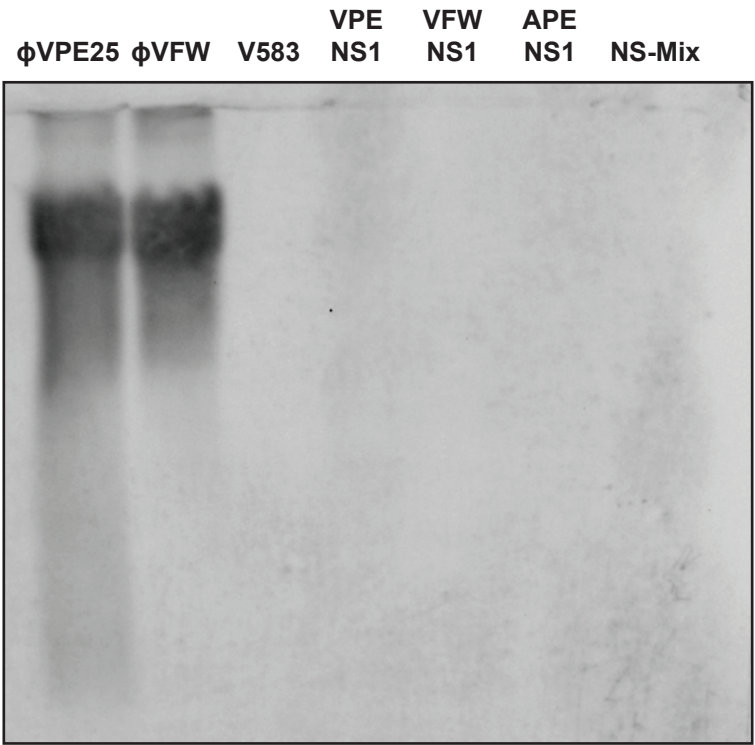

Supplement: Figure S2 — Phage-resistant E. faecalis isolates do not harbor integrated prophages. Genomic DNA isolated from E. faecalis cells that developed spontaneous resistance to φVPE25 and φVFW infection (see Table S2 in the supplemental material) lacks detectable phage DNA as determined by Southern blotting. The NS-mix lane contains a pool of E. faecalis V583 phage-resistant isolates recovered from scraping the soft agar of a semiconfluent φVPE25 lysis plate that was serially passaged in BHI three times prior to extraction of total genomic DNA for Southern blot analysis. The V583 lane contains genomic DNA from phage-sensitive wild-type E. faecalis V583. Purified φVPE25 and φVFW DNAs are included as controls. Download [file mbo004162963sf2.pdf]

Figure S3

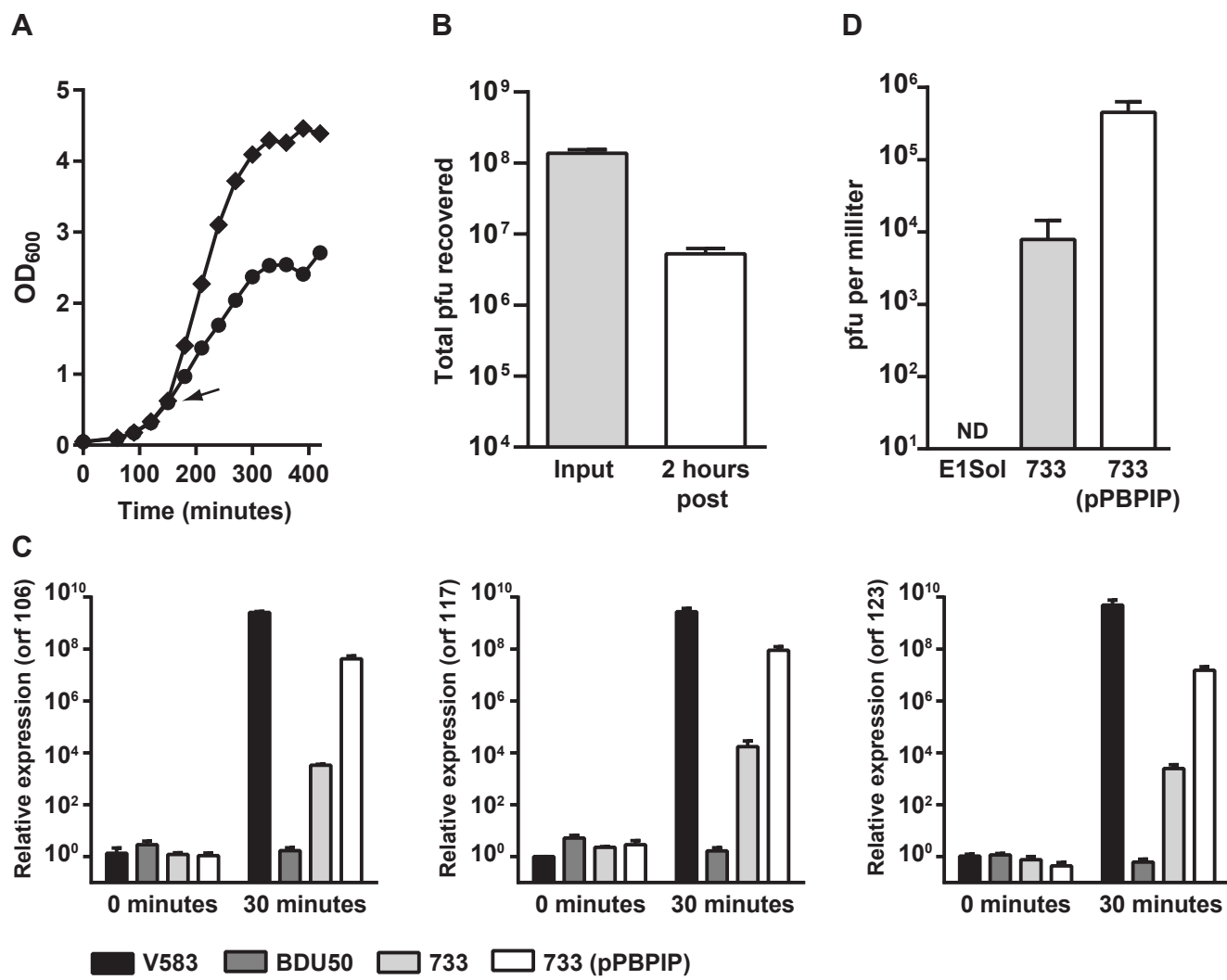

Supplement: Figure S3 — φVPE25 infection of transgenic E. faecium 1,141,733. (A) Growth kinetics of E. faecium 1,141,733 carrying the E. faecalis V583 PIPEF expression plasmid pPBPIP in the presence (●) and absence (♦) of φVPE25. The arrow indicates the time of φVPE25 addition to the culture. (B) φVPE25 particle numbers from infected E. faecalis V583 cells carrying pPBPIP immediately after phage addition (Input) or 2 h after phage infection (2 hours post). (C) Quantitative real-time PCR of the φVPE25 transcripts orf_106 (lysin), orf_117 (major tail protein), and orf_123 (major capsid protein) isolated from E. faecium 1,141,733 or E. faecium 1,141,733 carrying plasmid pPBPIP. E. faecalis V583 and the PIPEF mutant strain BDU50 are included as controls. Transcript abundances are plotted on a logarithmic scale. (D) Viable phage particles recovered from wild-type and PIPEF transgenic E. faecium 1,141,733 after cell disruption using lysozyme and sonication. E. faecalis E1Sol was included as a control strain that is resistant to φVPE25 infection. ND, none detected. Download [file mbo004162963sf3.pdf]

Figure S4

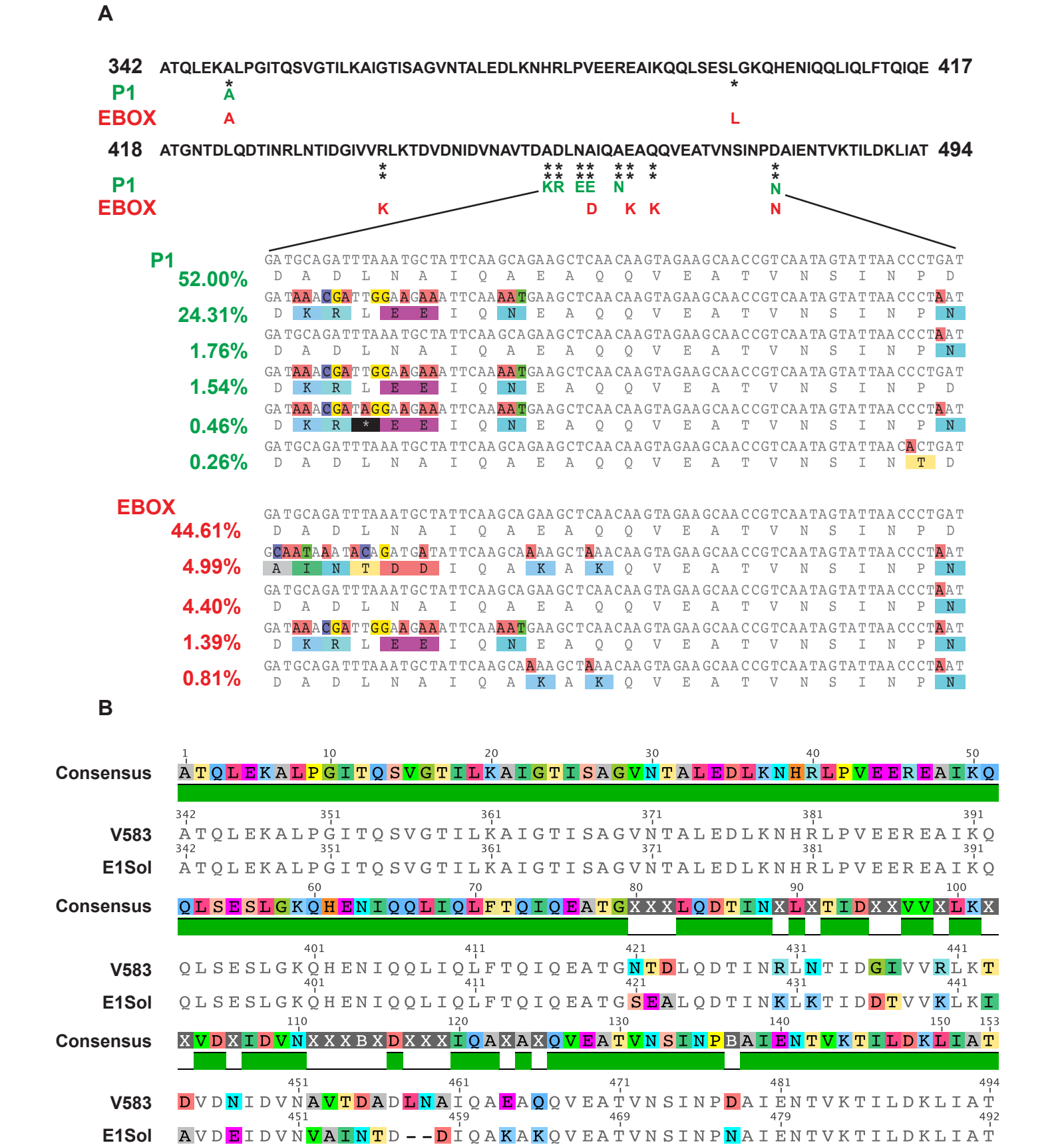

Supplement: Figure S4 — Sequence variation among the PIPEF variable regions of E. faecalis sewage isolates. (A) Schematic of the variable region of PIPEF (amino acids 342 to 494). The amino acids where variation was detected by direct PCR from raw sewage (EBOX) or from pooled enterococcal isolates grown on selective agar (P1) are indicated in red and green, respectively. For both EBOX and P1 samples, the majority of the amino acid content of the PIPEF variable region matched E. faecalis V583 (52.00% of contigs for P1 and 41.61% for EBOX). The top four or five representative contigs containing variant amino acid composition compared to the E. faecalis V583 PIPEF variable region sequence as a reference are indicated. (B) Alignment of the E. faecalis V583 (clade 4) and E1Sol (clade 5) PIPEF variable regions. Download [file mbo004162963sf4.pdf]
