# Supplementary material for: Molecular Basis for Lytic Bacteriophage Resistance in Enterococci
Source: mBio. 2016 Aug 30;7(4):e01304-16. doi: 10.1128/mBio.01304-16 (PMC4999554; doi:10.1128/mBio.01304-16)
Supplement: Table S1 — Enterococcal bacteriophage genome organization and features. [file mbo004162963st1.pdf]

Table S1. Enterococcal bacteriophage genome organization and features

ϕVPE25 genome annotation

Feature ID	Gene Start	Gene Stop	Strand	blastp Best-Hit cut-off (0.001)	Best-Hit Accesion Number	%Identity /Similarity /Gaps	E-value	RAST Function Prediction
orf_1	1033	1239	+	None				hypothetical protein
orf_2	1243	2841	+	None				hypothetical protein
orf_3	2927	4882	+	hypothetical protein [Weissella oryzae]	WP_027699 797	35/55/50	8.00E-105	RecD-like DNA helicase YrrC
orf_4	4860	5387	+	None				hypothetical protein
orf_5	5826	6137	+	None				hypothetical protein
orf_6	6134	6571	+	None				hypothetical protein
orf_7	6571	6825	+	None				hypothetical protein
orf_8	6828	7064	+	None				hypothetical protein
orf_9	7097	8383	+	ATP-dependent DNA ligase [Bacillus subtilis]	WP_020846 136	35/58/20	8.00E-68	DNA ligase, phage-associated
orf_10	8399	8590	+	None				hypothetical protein
orf_11	8753	8968	+	None				hypothetical protein
orf_12	9057	9260	+	None				hypothetical protein
orf_13	9281	9493	+	None				hypothetical protein
orf_14	9477	9887	+	None				hypothetical protein
orf_15	9984	10532	+	Rossmann fold nucleotide-binding protein [Enterococcus durans]	WP_005877 211	45/64/6	2.00E-42	Lysine decarboxylase family
orf_16	10529	10717	+	None				hypothetical protein
orf_17	10718	11344	+	nucleoside deoxyribosyltransferase [Lactococcus lactis]	WP_014573 106	49/66/4	7.00E-42	Purine trans deoxyribosylase (Nucleoside deoxyribosyltransferase-I) (EC 2.4.2.6)
orf_18	11435	12271	+	gp051 [Lactococcus phage KSY1]	YP_0014690 49	68/81/0	5.00E-130	Deoxyadenosine kinase (EC 2.7.1.76) / Deoxyguanosine kinase (EC 2.7.1.113)
orf_19	12293	13048	+	gp052 [Lactococcus phage KSY1]	YP_0014690 50	54/72/0	2.00E-92	NrdR-regulated deoxyribonucleotide transporter, PnuC-like
orf_20	13120	13398	+	None				hypothetical protein
orf_21	13402	13650	+	glutaredoxin [Mycobacterium abscessus]	WP_020724 515	43/60/1	8.00E-13	hypothetical protein
orf_22	13734	14771	+	hypothetical protein [Bacillus sp. YP1]	WP_043940 300	35/57/21	1.00E-46	hypothetical protein
orf_23	14831	15685	+	hypothetical protein THICB2_640135 [Thiomonas sp. CB2]	CDW95294	42/62/1	2.00E-08	hypothetical protein
orf_24	15678	16322	+	None				hypothetical protein
orf_25	16306	16473	+	None				hypothetical protein
orf_26	16574	17542	+	bacteriophage related protein [Bacillus subtilis]	WP_013603 304	42/60/26	1.00E-67	phage protein
orf_27	17625	18107	+	None				hypothetical protein

Feature ID	Gene Start	Gene Stop	Strand	blastp Best-Hit cut-off (0.001)	Best-Hit Accesion Number	%Identity /Similarity /Gaps	E-value	RAST Function Prediction
orf_28	18107	18535	+	None				hypothetical protein
orf_29	18606	19676	+	ATP/GTP binding protein [Bacillus subtilis]	WP_013603308	48/67/9	5.00E-110	ATP/GTP binding protein
orf_30	19781	20350	+	None				hypothetical protein
orf_31	20350	20607	+	None				hypothetical protein
orf_32	20667	20813	+	None				hypothetical protein
orf_33	20803	21543	+	None				hypothetical protein
orf_34	21583	21810	+	None				hypothetical protein
orf_35	21822	22064	+	hypothetical protein [Enterococcus faecalis]	WP_016622890	68/81/0	4.00E-14	hypothetical protein
orf_36	22077	22304	+	None				hypothetical protein
orf_37	22399	22611	+	hypothetical protein EFP_gp142 [Enterococcus phage phiEF24C]	YP_001504251	62/79/1	2.00E-20	Phage protein
orf_38	22626	23000	+	None				hypothetical protein
orf_39	23034	23537	+	bacteriophage related protein [Bacillus subtilis]	WP_013603315	41/64/6	3.00E-31	phage protein
orf_40	23534	25114	+	replicative DNA helicase [Bacillus subtilis]	WP_020846144	53/73/8	0	Replicative DNA helicase (DnaB) (EC 3.6.4.12)
orf_41	25126	26175	+	bacteriophage related protein [Bacillus subtilis]	WP_013603317	50/68/6	1.00E-111	Phage toprim domain containing protein, YorJ B.subtilis homolog
orf_42	26186	28012	+	single-stranded DNA exonuclease [Bacillus subtilis]	WP_020846146	40/57/30	2.00E-105	Single-stranded-DNA-specific exonuclease RecJ (EC 3.1.-.-)
orf_43	28109	28711	+	None				hypothetical protein
orf_44	28726	29109	+	None				hypothetical protein
orf_45	29102	29572	+	None				hypothetical protein
orf_46	29670	29798	+	None				hypothetical protein
orf_47	29881	30075	+	None				hypothetical protein
orf_48	30098	30244	+	None				hypothetical protein
orf_49	30246	30446	+	None				hypothetical protein
orf_50	30519	31034	+	None				hypothetical protein
orf_51	31028	31873	+	thymidylate synthase, partial [Rhizobium leguminosarum]	WP_032998484	38/57/2	3.00E-34	Thymidylate synthase (EC 2.1.1.45)
orf_52	31902	32318	+	MULTISPECIES: dUTP diphosphatase [Clostridiales]	WP_004083327	54/71/0	2.00E-44	Deoxyuridine 5'-triphosphate nucleotidohydrolase (EC 3.6.1.23)
orf_53	32328	33386	+	beta glucosyl transferase [Serratia phage PS2]	YP_009030087	42/63/10	3.00E-90	b-gt beta glucosyl transferase
orf_54	33458	33982	+	MULTISPECIES: ribonuclease HI [Lactobacillus]	WP_025013129	45/63/3	6.00E-37	Ribonuclease HI (EC 3.1.26.4)
orf_55	33979	34941	+	beta-1,6-galactofuranosyltransferase [Leuconostoc gelidum]	WP_010015365	21/58/18	2.00E-27	Nucleotide sugar synthetase-like protein
orf_56	34943	35188	+	None				hypothetical protein
orf_57	35181	35372	+	None				hypothetical protein
orf_58	35474	36082	+	thymidine kinase [Lactobacillus mali]	WP_010078319	45/62/11	4.00E-42	Thymidine kinase (EC 2.7.1.21)
orf_59	36084	36326	+	hypothetical protein T211_08215 [Lactococcus lactis subsp. lactis bv. diacetylactis str. LD61]	ESK79055	58/58/0	3.00E-08	hypothetical protein

Feature ID	Gene Start	Gene Stop	Strand	blastp Best-Hit cut-off (0.001)	Best-Hit Accesion Number	%Identity /Similarity /Gaps	E-value	RAST Function Prediction
orf_60	36337	36552	+	None				hypothetical protein
orf_61	36654	36842	+	None				hypothetical protein
orf_62	36925	40791	+	DNA polymerase III alpha subunit [Bacillus subtilis]	WP_020846153	47/67/33	0	DNA polymerase III alpha subunit (EC 2.7.7.7)
orf_63	40861	41115	+	None				hypothetical protein
orf_64	41115	41384	+	None				hypothetical protein
orf_65	41693	41884	+	None				hypothetical protein
orf_66	41896	42204	+	None				hypothetical protein
orf_67	42194	42739	+	None				hypothetical protein
orf_68	42741	43028	+	None				hypothetical protein
orf_69	43045	43323	+	None				hypothetical protein
orf_70	43387	43623	+	hypothetical protein [Bacillus thuringiensis]	WP_001009047	51/64/7	3.00E-14	hypothetical protein
orf_71	43626	44327	+	dUTP diphosphatase [Enterococcus villorum]	WP_010750123	45/56/61	8.00E-50	Deoxyuridine 5'-triphosphate nucleotidohydrolase (EC 3.6.1.23)
orf_72	44327	44620	+	None				hypothetical protein
orf_73	44617	45240	+	guanylate kinase/L-type calcium channel region [Bacillus subtilis]	WP_009968548	37/56/11	7.00E-29	Guanylate kinase (EC 2.7.4.8)
orf_74	45240	45815	+	rusA family endodeoxyribonuclease [Bacillus subtilis]	WP_013603335	39/61/1	9.00E-36	non-essential protein
orf_75	45796	46125	+	None				hypothetical protein
orf_76	46206	46748	+	None				hypothetical protein
orf_77	46978	47127	+	None				hypothetical protein
orf_78	47230	47577	+	None				hypothetical protein
orf_79	47658	48293	+	None				hypothetical protein
orf_80	48307	48504	+	None				hypothetical protein
orf_81	48501	48704	+	None				hypothetical protein
orf_82	48705	48911	+	None				hypothetical protein
orf_83	48960	50900	+	subunit B of ATP-hydrolyzing DNA topoisomerase [Bacillus subtilis]	WP_013603341	50/69/22	0	Topoisomerase IV subunit B (EC 5.99.1.-)
orf_84	50983	51192	+	None				hypothetical protein
orf_85	51193	53178	+	DNA gyrase/topoisomerase IV, subunit A [Bacillus subtilis]	WP_009968555	40/61/28	7.00E-154	Topoisomerase IV subunit A (EC 5.99.1.-)
orf_86	53329	53478	+	None				hypothetical protein
orf_87	53558	53746	+	None				hypothetical protein
orf_88	53921	54640	+	Aggregation promoting factor [Catellicoccus marimammalium]	WP_009490292	54/63/2	6.00E-33	Aggregation promoting factor
orf_89	54772	54975	+	None				hypothetical protein
orf_90	55083	55289	+	hypothetical protein EfaCPT1_gp47 [Enterococcus phage EfaCPT1]	YP_009103864	97/97/0	2.00E-39	hypothetical protein
orf_91	55283	55600	+	hypothetical protein CO47_0131 [Enterococcus phage AUEF3]	AHN83302	96/99/0	3.00E-65	hypothetical protein
orf_92	55640	55807	+	hypothetical protein EFP_gp137 [Enterococcus phage phiEF24C]	YP_001504246	80/90/0	2.00E-19	Phage protein
orf_93	55902	56075	+	hypothetical protein HMPREF9087_1257 [Enterococcus casseliflavus ATCC 12755]	EGC69869	44/68/0	6.00E-04	hypothetical protein

Feature ID	Gene Start	Gene Stop	Strand	blastp Best-Hit cut-off (0.001)	Best-Hit Accesion Number	%Identity /Similarity /Gaps	E-value	RAST Function Prediction
orf_94	56076	56279	+	None				hypothetical protein
orf_95	56280	56540	+	None				hypothetical protein
orf_96	56541	56753	+	None				hypothetical protein
orf_97	56754	57161	+	hypothetical protein EnPhEFRM31_gp06 [Enterococcus phage EFRM31]	YP_004306634	58/77/1	4.00E-47	Phage tail length tape-measure protein
orf_98	57194	57400	+	None				hypothetical protein
orf_99	57390	57794	+	None				hypothetical protein
orf_100	57787	57993	+	None				hypothetical protein
orf_101	58416	58607	+	None				hypothetical protein
orf_102	58642	58854	+	None				hypothetical protein
orf_103	58930	59094	+	None				hypothetical protein
orf_104	59094	59651	+	None				hypothetical protein
orf_105	60672	59695	-	site specific recombinase [Bacillus subtilis]	WP_013603353	64/82/0	3.00E-150	prophage LambdaBa02, site-specific recombinase, phage integrase family
orf_106	61881	60739	-	lysin [Enterococcus phage IME-EF4]	YP_009004353	53/64/28	4.00E-115	N-acetylmuramoyl-L-alanine amidase (EC 3.5.1.28)
orf_107	62327	61962	-	lysis protein [Lactobacillus fabifermentans]	WP_033613708	40/57/2	8.00E-04	hypothetical protein
orf_108	62686	62342	-	None				hypothetical protein
orf_109	63062	62664	-	None				hypothetical protein
orf_110	64679	63108	-	hypothetical protein [Enterococcus faecalis]	WP_025193464	32/45/103	5.00E-61	hypothetical protein
orf_111	66627	64699	-	hypothetical protein WOC_00151 [Enterococcus faecalis EnGen0357]	EOJ87326	29/47/104	1.00E-65	hypothetical protein
orf_112	69221	66627	-	phage minor structural protein [Enterococcus malodoratus]	WP_010738973	49/69/18	1.00E-163	FIG00629079: hypothetical protein
orf_113	70012	69218	-	phage tail protein [Enterococcus faecalis]	WP_023895112	48/65/5	5.00E-78	hypothetical protein
orf_114	73818	70012	-	tail protein [Enterococcus faecium]	WP_002318243	33/53/122	0	Phage tail length tape-measure protein
orf_115	74097	73846	-	None				hypothetical protein
orf_116	74404	74072	-	hypothetical protein [Eubacterium acidaminophilum]	WP_025436582	38/59/4	4.00E-09	hypothetical protein
orf_117	75147	74530	-	phage major tail protein, phi13 family [Brevibacillus agri]	WP_005826684	44/60/2	4.00E-45	Phage major tail protein phi13
orf_118	75550	75173	-	None				hypothetical protein
orf_119	76002	75553	-	hypothetical protein [Geobacillus vulcani]	WP_031405270	33/53/25	2.00E-08	hypothetical protein
orf_120	76357	75995	-	hypothetical protein [Veillonella sp. CAG:933]	WP_021841991	35/50/2	4.00E-08	hypothetical protein
orf_121	76758	76354	-	head-tail connector protein [Staphylococcus phage vB_SepS_SEP9]	YP_009007676	39/60/6	3.00E-18	hypothetical protein

Feature ID	Gene Start	Gene Stop	Strand	blastp Best-Hit cut-off (0.001)	Best-Hit Accesion Number	%Identity /Similarity /Gaps	E-value	RAST Function Prediction
orf_122	77787	76915	-	hypothetical protein [Lactobacillus paracasei]	WP_025376065	49/67/11	2.00E-96	prophage pi2 protein 34
orf_123	79238	78036	-	major capsid protein [Lactobacillus phage ATCC 8014-B2]	AFU63091	41/57/41	6.00E-91	Gene Transfer Agent capsid protein; Phage major capsid protein
orf_124	80406	79228	-	phage major capsid protein [Bacillus subtilis]	WP_020846131	45/66/10	6.00E-41	Phage head maturation protease
orf_125	81713	80433	-	phage portal protein, HK97 family [Bacillus subtilis]	WP_009968694	44/62/16	2.00E-108	Phage portal protein
orf_126	83436	81730	-	terminase [Bacillus sp. YP1]	WP_043940336	64/80/7	0	Phage terminase, large subunit
orf_127	83510	83626	+	None				hypothetical protein
orf_128	84017	83748	-	None				hypothetical protein
orf_129	84674	84183	-	small subunit of P27 family phage terminase [Bacillus subtilis]	WP_013603375	60/76/0	3.00E-64	Phage-related protein
orf_130	85094	84687	-	HNH restriction endonuclease domain protein [Bacillus subtilis]	WP_020846133	53/69/2	8.00E-37	hypothetical protein
orf_131	85376	85531	+	None				hypothetical protein
orf_132	85521	86381	+	None				hypothetical protein

ϕVFW genome annotation

Feature ID	Gene Start	Gene Stop	Strand	blastp Best-Hit cut-off (0.001)	Best-Hit Accesion Number	%Identity /Similarity /Gaps	E-value	RAST Function Prediction
orf_1	953	1159	+	None				hypothetical protein
orf_2	1163	2761	+	None				hypothetical protein
orf_3	2847	4811	+	hypothetical protein [Weissella oryzae]	WP_027699797	36/55/44	1.00E-104	RecD-like DNA helicase YrrC
orf_4	4822	5316	+	None				hypothetical protein
orf_5	5524	5646	+	None				hypothetical protein
orf_6	5756	6067	+	None				hypothetical protein
orf_7	6064	6501	+	None				hypothetical protein
orf_8	6501	6755	+	None				hypothetical protein
orf_9	6758	6994	+	None				hypothetical protein
orf_10	7027	8313	+	hypothetical protein [Bacillus sp. YP1]	WP_043940297	35/58/20	3E-68	DNA ligase, phage-associated
orf_11	8329	8520	+	None				hypothetical protein
orf_12	8684	8899	+	None				hypothetical protein
orf_13	8990	9193	+	None				hypothetical protein
orf_14	9214	9426	+	None				hypothetical protein
orf_15	9410	9820	+	None				hypothetical protein
orf_16	9917	10465	+	Rossmann fold nucleotide-binding protein [Enterococcus durans]	WP_005877211	45/63/6	5E-42	Lysine decarboxylase family
orf_17	10462	10650	+	None				hypothetical protein
orf_18	10651	11277	+	nucleoside deoxyribosyltransferase [Lactococcus lactis]	WP_014573106	49/65/4	5.00E-41	Purine trans deoxyribosylase (Nucleoside deoxyribosyltransferase-I) (EC 2.4.2.6)
orf_19	11368	12204	+	gp051 [Lactococcus phage KSY1]	YP_001469049	68/81/0	6.00E-130	Deoxyadenosine kinase (EC 2.7.1.76) / Deoxyguanosine kinase (EC 2.7.1.113)
orf_20	12226	12981	+	gp052 [Lactococcus phage KSY1]	YP_001469050	53/72/0	7E-92	NrdR-regulated deoxyribonucleotide transporter, PnuC-like
orf_21	13053	13331	+	None				hypothetical protein
orf_22	13335	13583	+	glutaredoxin [Mycobacterium abscessus]	WP_020724515	43/59/5	8.00E-13	hypothetical protein
orf_23	13667	14704	+	hypothetical protein [Bacillus sp. YP1]	WP_043940300	35/56/21	1.00E-46	hypothetical protein
orf_24	14764	15618	+	hypothetical protein THICB2_640135 [Thiomonas sp. CB2]	CDW95294	42/62/1	0.00000002	hypothetical protein
orf_25	15611	16255	+	None				hypothetical protein
orf_26	16239	16406	+	None				hypothetical protein
orf_27	16507	17475	+	bacteriophage related protein [Bacillus subtilis]	WP_013603304	42/60/26	1E-67	phage protein
orf_28	17558	18037	+	None				hypothetical protein

Feature ID	Gene Start	Gene Stop	Strand	blastp Best-Hit cut-off (0.001)	Best-Hit Accesion Number	%Identity /Similarity /Gaps	E-value	RAST Function Prediction
orf_29	18037	18465	+	None				hypothetical protein
orf_30	18539	19609	+	ATP/GTP binding protein [Bacillus subtilis]	WP_013603308	47/67/9	3.00E-109	ATP/GTP binding protein
orf_31	19713	20282	+	None				hypothetical protein
orf_32	20282	20539	+	None				hypothetical protein
orf_33	20536	20745	+	None				hypothetical protein
orf_34	20735	21475	+	hypothetical protein [Paenibacillus pinihumi]	WP_036708774.1	39/59/0	0.00006	hypothetical protein
orf_35	21515	21742	+	None				hypothetical protein
orf_36	21754	21996	+	hypothetical protein [Enterococcus faecalis]	WP_016622890	68/81/0	4.00E-14	hypothetical protein
orf_37	22009	22236	+	None				hypothetical protein
orf_38	22331	22543	+	hypothetical protein EFP_gp142 [Enterococcus phage phiEF24C]	YP_001504251	62/79/1	2.00E-20	Phage protein
orf_39	22558	22932	+	None				hypothetical protein
orf_40	22966	23469	+	bacteriophage related protein [Bacillus subtilis]	WP_013603315	41/64/6	3.00E-31	phage protein
orf_41	23466	25046	+	hypothetical protein [Bacillus sp. YP1]	WP_043940316	53/73/8	0	Replicative DNA helicase (DnaB) (EC 3.6.4.12)
orf_42	25058	26107	+	hypothetical protein [Bacillus subtilis]	WP_009968513	50/68/6	7.00E-112	Phage toprim domain containing protein, YorJ B.subtilis homolog
orf_43	26118	27944	+	single-stranded DNA exonuclease [Bacillus subtilis]	WP_020846146	40/57/30	2.00E-105	Single-stranded-DNA-specific exonuclease RecJ (EC 3.1.-.-)
orf_44	28041	28643	+	None				hypothetical protein
orf_45	28658	29041	+	None				hypothetical protein
orf_46	29034	29504	+	None				hypothetical protein
orf_47	29602	29730	+	None				hypothetical protein
orf_48	29813	30007	+	None				hypothetical protein
orf_49	30030	30176	+	None				hypothetical protein
orf_50	30178	30378	+	None				hypothetical protein
orf_51	30451	30966	+	None				hypothetical protein
orf_52	30960	31805	+	hypothetical protein [Chryseobacterium luteum]	WP_034707540	36/60/8	7.00E-37	Thymidylate synthase (EC 2.1.1.45)
orf_53	31834	32250	+	dUTP diphosphatase [Clostridiales]	WP_004083327	54/71/0	2.00E-44	Deoxyuridine 5'-triphosphate nucleotidohydrolase (EC 3.6.1.23)
orf_54	32260	33318	+	beta glucosyl transferase [Serratia phage PS2]	YP_009030087	42/63/10	4.00E-90	b-gt beta glucosyl transferase
orf_55	33390	33914	+	ribonuclease HI [Lactobacillus]	WP_025013129	45/65/3	6.00E-37	Ribonuclease HI (EC 3.1.26.4)
orf_56	33911	34873	+	hypothetical protein, partial [Leuconostoc citreum]	WP_036069825	30/50/43	2.00E-29	Nucleotide sugar synthetase-like protein
orf_57	34875	35120	+	None				hypothetical protein
orf_58	35113	35304	+	None				hypothetical protein
orf_59	35406	36014	+	thymidine kinase [Lactobacillus mali]	WP_010078319.1	45/62/5	5.00E-43	Thymidine kinase (EC 2.7.1.21)

Feature ID	Gene Start	Gene Stop	Strand	blastp Best-Hit cut-off (0.001)	Best-Hit Accesion Number	%Identity /Similarity /Gaps	E-value	RAST Function Prediction
orf_60	36016	36258	+	hypothetical protein T211_08215 [Lactococcus lactis subsp. lactis bv. diacetylactis str. LD61]	ESK79055	58/58/0	3.00E-09	hypothetical protein
orf_61	36269	36484	+	None				hypothetical protein
orf_62	36585	36773	+	None				hypothetical protein
orf_63	36856	40722	+	DNA polymerase III alpha subunit [Bacillus subtilis]	WP_020846153	47/67/33	0	DNA polymerase III alpha subunit (EC 2.7.7.7)
orf_64	40792	41046	+	None				hypothetical protein
orf_65	41046	41315	+	None				hypothetical protein
orf_66	41625	41816	+	None				hypothetical protein
orf_67	41828	42136	+	None				hypothetical protein
orf_68	42126	42671	+	None				hypothetical protein
orf_69	42673	42960	+	None				hypothetical protein
orf_70	42977	43255	+	None				hypothetical protein
orf_71	43319	43555	+	hypothetical protein [Bacillus thuringiensis]	WP_001009047	51/64/7	3E-14	hypothetical protein
orf_72	43558	44259	+	dUTP diphosphatase [Enterococcus villorum]	WP_010750123	45/56/61	8.00E-50	Deoxyuridine 5'-triphosphate nucleotidohydrolase (EC 3.6.1.23)
orf_73	44259	44552	+	None				hypothetical protein
orf_74	44549	45172	+	guanylate kinase/L-type calcium channel region [Bacillus subtilis]	WP_009968548	37/56/11	3E-28	Guanylate kinase (EC 2.7.4.8)
orf_75	45172	45747	+	rusA family endodeoxyribonuclease [Bacillus subtilis]	WP_013603335	39/61/1	9.00E-36	non-essential protein
orf_76	45728	46057	+	None				hypothetical protein
orf_77	46138	46680	+	None				hypothetical protein
orf_78	46909	47058	+	None				hypothetical protein
orf_79	47162	47509	+	None				hypothetical protein
orf_80	47590	48225	+	RNA polymerase subunit sigma-70 [Bacillus subtilis]	WP_009968495	29/55/18	4E-17	hypothetical protein
orf_81	48240	48449	+	None				hypothetical protein
orf_82	48446	48655	+	None				hypothetical protein
orf_83	48705	48866	+	None				hypothetical protein
orf_84	48867	50807	+	DNA topoisomerase [Bacillus subtilis]	WP_009968556	49/69/22	0	Topoisomerase IV subunit B (EC 5.99.1.-)
orf_85	50890	51099	+	None				hypothetical protein
orf_86	51100	53085	+	hypothetical protein, partial [Bacillus sp. YP1]	WP_043940343	41/61/24	3E-153	Topoisomerase IV subunit A (EC 5.99.1.-)
orf_87	53236	53385	+	None				hypothetical protein
orf_88	53465	53653	+	None				hypothetical protein
orf_89	53831	54550	+	Aggregation promoting factor [Catellicoccus marimammalium]	WP_009490292	54/64/2	9E-34	Aggregation promoting factor
orf_90	54676	54879	+	None				hypothetical protein
orf_91	54985	55293	+	hypothetical protein EfaCPT1_gp58 [Enterococcus phage EfaCPT1]	YP_009103875	78/83/0	7E-48	Phage protein
orf_92	55306	55476	+	hypothetical protein EFP_gp137 [Enterococcus phage phiEF24C]	YP_001504246	80/90/0	6.00E-20	Phage protein
orf_93	55489	55701	+	None				hypothetical protein

Feature ID	Gene Start	Gene Stop	Strand	blastp Best-Hit cut-off (0.001)	Best-Hit Accesion Number	%Identity /Similarity /Gaps	E-value	RAST Function Prediction
orf_94	55859	56035	+	None				hypothetical protein
orf_95	56032	56424	+	None				hypothetical protein
orf_96	56455	56667	+	None				hypothetical protein
orf_97	56657	57061	+	None				hypothetical protein
orf_98	57054	57260	+	None				hypothetical protein
orf_99	57685	57876	+	None				hypothetical protein
orf_100	57911	58123	+	None				hypothetical protein
orf_101	58198	58362	+	None				hypothetical protein
orf_102	58362	58919	+	None				hypothetical protein
orf_103	59942	58965	-	hypothetical protein [Bacillus sp. YP1]	WP_043940287	64/82/0	2E-150	prophage LambdaBa02, site-specific recombinase, phage integrase family
orf_104	61151	60009	-	lysin [Enterococcus phage IME-EF4]	YP_009004353	53/64/28	5E-115	N-acetylmuramoyl-L-alanine amidase (EC 3.5.1.28)
orf_105	61597	61232	-	lysis protein [Lactobacillus fabifermentans]	WP_033613708	40/57/2	0.0008	hypothetical protein
orf_106	61956	61612	-	hypothetical protein [Listeria rocourtiae]	WP_036069146.1	35/58/0	0.0002	hypothetical protein
orf_107	62332	61934	-	None				hypothetical protein
orf_108	63946	62378	-	hypothetical protein [Enterococcus faecalis]	WP_025193464	31/46/105	3.00E-60	hypothetical protein
orf_109	65894	63966	-	hypothetical protein WOC_00151 [Enterococcus faecalis EnGen0357]	EOJ87326	29/47/92	5.00E-68	hypothetical protein
orf_110	68487	65908	-	phage minor structural protein, partial [Enterococcus faecalis]	WP_033656155	49/67/14	5.00E-165	Phage endopeptidase
orf_111	69278	68484	-	phage tail protein [Enterococcus faecalis]	WP_023895112	48/65/5	1E-77	hypothetical protein
orf_112	73084	69278	-	tail protein [Enterococcus faecium]	WP_002318243	33/53/122	0	Phage tail length tape-measure protein
orf_113	73363	73112	-	None				hypothetical protein
orf_114	73670	73338	-	hypothetical protein [Eubacterium acidaminophilum]	WP_025436582	38/59/4	4.00E-09	hypothetical protein
orf_115	74412	73795	-	phage major tail protein, phi13 family [Brevibacillus agri]	WP_005826684	44/60/2	2.00E-45	Phage major tail protein phi13
orf_116	74815	74438	-	None				hypothetical protein
orf_117	75267	74818	-	hypothetical protein [Geobacillus vulcani]	WP_031405270	33/53/25	0.00000002	hypothetical protein
orf_118	75622	75260	-	hypothetical protein [Veillonella sp. CAG:933]	WP_021841991	35/50/2	0.00000004	hypothetical protein
orf_119	76023	75619	-	head-tail connector protein [Staphylococcus phage vB_SepS_SEP9]	YP_009007676	38/60/6	2.00E-18	hypothetical protein
orf_120	77067	76180	-	hypothetical protein [Lactobacillus paracasei]	WP_025376065	49/67/11	1.00E-96	prophage pi2 protein 34
orf_121	78567	77365	-	major capsid protein [Lactobacillus phage ATCC 8014-B2]	AFU63091	41/57/41	6E-91	Gene Transfer Agent capsid protein; Phage major capsid protein #Fam0006

Feature ID	Gene Start	Gene Stop	Strand	blastp Best-Hit cut-off (0.001)	Best-Hit Accesion Number	%Identity /Similarity /Gaps	E-value	RAST Function Prediction
orf_122	79735	78557	-	phage major capsid protein [Bacillus subtilis]	WP_020846 131	45/66/10	4.00E-41	Phage head maturation protease
orf_123	81042	79762	-	phage portal protein, HK97 family [Bacillus subtilis]	WP_009968 694	44/62/16	2.00E-108	Phage portal protein
orf_124	82765	81059	-	terminase [Bacillus sp. YP1]	WP_043940 336	64/80/7	0.00E+00	Phage terminase, large subunit
orf_125	82839	82955	+	None				hypothetical protein
orf_126	83346	83077	-	hypothetical protein, partial [Enterococcus faecalis]	WP_033918 705	65/73/5	2E-27	hypothetical protein
orf_127	84003	83512	-	small subunit of P27 family phage terminase [Bacillus subtilis]	WP_013603 375	60/76/0	3.00E-64	Phage-related protein
orf_128	84423	84016	-	hypothetical protein [Bacillus subtilis]	WP_013603 376	54/70/3	2.00E-38	hypothetical protein
orf_129	84705	84860	+	None				hypothetical protein
orf_130	84850	85710	+	None				hypothetical protein

Coding Sequence Annotation

Replication/Biosynthesis

DNA restriction modification

Host cell Lysis

Structural morphogenesis

DNA packaging

Hypothetical
