# Supplementary material for: Molecular Basis for Lytic Bacteriophage Resistance in Enterococci
Source: mBio. 2016 Aug 30;7(4):e01304-16. doi: 10.1128/mBio.01304-16 (PMC4999554; doi:10.1128/mBio.01304-16)
Supplement: Table S2 — Spontaneous mutations in EF0858 (PIPEF) result in phage resistance. [file mbo004162963st2.pdf]

Table S2. Spontaneous mutations in EF0858 (PIP_{EF}) result in phage resistance.

Phage used for isolation	Clone	Reference position	Variation type	Reference	Variation	Variation freq. (%)	Fold coverage	Amino acid change	ϕVPE25 and ϕVFW resistance
ϕVPE25	APENS1	815,579	ISE		5’ insertion	-	-	Frameshift	+
	APENS2	816,873 – 816,882	DIP	ATACGATTGA	-	100	509	Frameshift	+
	APENS3	817,761	DIP	C	-	100	417	Frameshift	+
	VPENS1	816,873 – 816,882	DIP	ATACGATTGA	-	100	546	Frameshift	+
	VPENS2	816,600	DIP	G	-	100	539	Frameshift	+
	VPENS3	816,785	SNP	G	T	98.9	471	Glu → Stop	+
ϕVFW	VFWNS1	816,873 – 816,882	DIP	ATACGATTGA	-	100	606	Frameshift	+
	VFWNS2	815,741	SNP	G	T	99.6	263	Gly → Stop	+
	VFWNS3	815,741	SNP	G	T	100	412	Gly → Stop	+

ISE – Insertion sequence element, DIP – Deletion or insertion polymorphism, SNP – Single nucleotide polymorphism
