# Supplementary material for: Molecular Basis for Lytic Bacteriophage Resistance in Enterococci
Source: mBio. 2016 Aug 30;7(4):e01304-16. doi: 10.1128/mBio.01304-16 (PMC4999554; doi:10.1128/mBio.01304-16)
Supplement: Table S3 — Sewage PIP read mapping to clade-specific PIPEF variable region. [file mbo004162963st3.pdf]

Table S3. Sewage PIP read mapping to clade specific PIP_{EF} variable region

Clade specific read matches*	Sample (% of reads mapped) [average fold coverage]			
	V583	EBOX	P1	P2
ARO1/DG - Clade 1	(0.99) [27.68]	(1.83) [75.08]	(12.30) [495.30]	(23.96) [994.39]
Merz96 - Clade 2	(0.61) [14.10]	(1.61) [44.27]	(0.89) [27.47]	(0.31) [7.47]
OG1RF - Clade 3	(0.15) [1.87]	(0.21) [5.12]	(0.16) [2.35]	(0.09) [1.48]
V583 - Clade 4	(76.98) [3033.77]	(25.72) [1536.97]	(46.25) [1883.98]	(49.37) [2062.34]
E1Sol - Clade 5	(16.61) [609.69]	(67.19) [4185.29]	(22.27) [864.08]	(7.46) [285.61]
Unmatched reads	4.66	3.89	18.12	18.80

* Indicates only one representative member from each clade was used for the read mapping
