# Supplementary material for: Molecular Basis for Lytic Bacteriophage Resistance in Enterococci
Source: mBio. 2016 Aug 30;7(4):e01304-16. doi: 10.1128/mBio.01304-16 (PMC4999554; doi:10.1128/mBio.01304-16)
Supplement: Table S4 — Mutations conferring φVPE25 resistance in E. faecalis from gnotobiotic mouse feces. [file mbo004162963st4.pdf]

Table S4. Mutations conferring ϕVPE25 resistance in *E. faecalis* from gnotobiotic mouse feces

Class of mutation	Position*	Reference	Variation	Result of mutation
Nonsense (35†)	815718	G	A	W → stop codon
	817799	C	T	Q → stop codon
	816467	G	T	E → stop codon
	815622	G	A	W → stop codon
	816491	C	T	Q → stop codon
	817073	C	T	Q → stop codon
	817419	T	A	L → stop codon
Deletion/Insertion (25†)	815754	C	Deletion	Frameshift
	816095	A	Deletion	Frameshift
	818140	G	Deletion	Frameshift
	816467	G	Deletion	Frameshift
	815619-815620	AT	Insertion, AAT	Frameshift
Transposon insertion (20†)	815641	N/A	IS256 insertion	Frameshift
	815661	N/A	IS256 insertion	Frameshift
	815610	N/A	IS256 insertion	Frameshift
	816130	N/A	IS216 insertion	Frameshift

*Position corresponds to genome coordinates of *E. faecalis* V583. † Percent frequency of the class of mutation
