# Supplementary material for: Molecular Basis for Lytic Bacteriophage Resistance in Enterococci
Source: mBio. 2016 Aug 30;7(4):e01304-16. doi: 10.1128/mBio.01304-16 (PMC4999554; doi:10.1128/mBio.01304-16)
Supplement: Table S5 — Bacterial strains, phages, plasmids, and primers used in this study. [file mbo004162963st5.pdf]

Table S5. Bacterial strains, phages, plasmids and primers.

Strains, phages, plasmids and primers	Characteristics and/or description	Reference/source
Enterococcus faecalis		
V583	Human blood isolate; Vm^R, Em^R, Gm^R	(1)
ATCC29212	Human urine isolate; Quality control strain; Tc^R	(2)
T1	SS498; CDC reference strain,	(3)
T2	Human urine isolate; Sapporo-603; Tc^R	(3)
T3	Human urine isolate; Sapporo-109; Tc^R	(3)
T8	Human urine isolate; Nagasaki-742; Tc^R, Em^R	(3)
T11	Human urine isolate; Sapporo-027;	(3)
X98	Infant fecal isolate; Lancefield H69D6	(3)
Fly1	Commensal isolate from Drosophila	(3)
AR01/DG	Dog wound isolate; Vm^R, Tc^R, Em^R	(3)
DS5	FDA strain PCI1326, Tc^R, Em^R	(3)
JH1	Clinical isolate; Tc^R, Em^R, Gm^R	(3)
D6	Pig isolate; Tc^R, Em^R, Gm^R	(3)
ATCC4200	Human blood isolate; Patient with rheumatic fever	(3)
CH188	Human liver isolate; Tc^R, Em^R, Gm^R, Cm^R, Ap^R	(3)
E1Sol	Human fecal isolate; Solomon Islands	(3)
HIP11704	Clinical isolate; Vm^R, Em^R, Gm^R	(3)
OG1RF	Human oral isolate; Rf^R, Fa^R	(4)
Merz96	Human blood isolate; Vm^R, Tc^R, Em^R, Gm^R	(3)
BDU50	ΔEF0858 PIP$_{EF}$ mutant of V583	This study
BDU51	PIP$_{Ef}$ replacement strain; EFJG_01790 with EF0858 in E1Sol	This study
BDU53	EF0858 PIP$_{EF}$ variable region mutant in V583; Δaa 342-390	This study
BDU54	EF0858 PIP$_{EF}$ variable region mutant in V583; Δaa 391-439	This study
BDU55	EF0858 PIP$_{EF}$ variable region mutant in V583; Δaa 440-494	This study
APENS1	Phage resistant mutant of V583; PIP$_{EF}$ 5' ISE	This study
APENS2	Phage resistant mutant of V583; PIP$_{EF}$ ATACGATTGA insertion	This study
APENS3	Phage resistant mutant of V583; PIP$_{EF}$ nucleotide deletion frameshift	This study
VPENS1	Phage resistant mutant of V583; PIP$_{EF}$ ATACGATTGA insertion	This study
VPENS2	Phage resistant mutant of V583; PIP$_{EF}$ nucleotide deletion frameshift	This study
VPENS3	Phage resistant mutant of V583; PIP$_{EF}$ nucleotide change; Glu to Stop	This study
VFWNS1	Phage resistant mutant of V583; PIP$_{EF}$ ATACGATTGA insertion	This study
VFWNS2	Phage resistant mutant of V583; PIP$_{EF}$ nucleotide change; Gly to Stop	This study
VFWNS3	Phage resistant mutant of V583; PIP$_{EF}$ nucleotide change; Gly to stop	This study
Enterococcus faecium		
Com12	Human fecal isolate	(3)
Com15	Human fecal isolate	(3)
1,141,733	Human blood isolate	(3)
Escherichia coli		
TG1	[F' *traD36 proAB lacIqZ* Δ*M15*] *supE thi-1* Δ(*lac-proAB*) Δ(*mcrB-hsdSM*)5(*rK - mK -*)	Lucigen
Phages		
ϕVPE25	*Siphoviridae*; Wastewater isolate	This study
ϕVFW	*Siphoviridae*; Wastewater isolate	This study
Plasmids		
pLT06	*E. faecalis* allelic exchange vector; Cm^R	(5)
pLZ12	Shuttle vector; pSH71 origin; Cm^R	(6)
pAT28-cat	Shuttle vector; pUC and pAMβ1 origins; Cm^R	Rodrigues and Palmer, 2016
pLTPIP	pLT06 with ΔEF0858 extending from +25 to +2649 bp with respect to the predicted start site; Cloned as a PstI-NcoI fragment; Allelic exchange vector for deletion of PIP$_{EF}$ in *E. faecalis* V583	This study
pLTDPIPA	pLT06 with ΔEF0858 extending from +1024 to +1170 bp with respect to the predicted start site; Cloned as a XbaI-NcoI fragment; Allelic	This study

	exchange vector for ΔA truncation of PIP_{EF} in *E. faecalis* V583	
pLTDPIPB	pLT06 with ΔEF0858 extending from +1171 to +1317 bp with respect to the predicted start site; Clone as a XbaI-NcoI fragment; Allelic exchange vector for ΔB truncation of PIP_{EF} in *E. faecalis* V583	This study
pLTDPIPC	pLT06 with ΔEF0858 extending from +1318 to +1482 bp with respect to the predicted start site; Cloned as a XbaI-NcoI fragment; Allelic exchange vector for ΔC truncation of PIP_{EF} in *E. faecalis* V583	This study
pLTPdV1	pLT06 with EF0858 from +76 bp to +2751 with respect to the predicted start site; Cloned as a XbaI-NcoI fragment; Allelic replacement vector used to generate transgenic PIP_{EF} in *E. faecalis* E1Sol	This study
pLZPIP	pLZ12 carrying V583 PIP_{EF} from -180 to +2780 with respect to the predicted start site; Complementation plasmid; Cloned as a PstI-BglII fragment	This study
pLZEV	pLZ12 carrying chimeric PIP_{EF}; E1sol PIP_{Ef} from -180 to +1261 fused to V583 PIP_{Ef} from +1262 to +2769 bp with respect to the predicted start site; PIP swapping plasmid for *E. faecalis*; Cloned as a PstI-BglII fragment	This study
pPBPIP	pAT28-cat carrying V583 PIP_{EF} from -180 to +2780 with respect to the predicted start site; PIP swapping plasmid for *E. faecium*; Cloned as a KpnI fragment	This study
Primers		
EF0858L-F	NNNNNNCTGCAGGATCAACAGAAACTGGTAGAGGACTATC; Forward primer for the left homology fragment and for final overlap extension PCR to generate pLTPIP; PstI site	This study
EF0858L-R	CAGCTTTTTGTTTTTTAATGGAATAAATGGCTCCATGTATTTTTATATGTTTCATAAAATCACCTGC; Reverse primer for the left homology fragment used to generate pLTPIP	This study
EF0858R-F	GCAGGTGATTTTATGAAACATATAAAAAATACATGGAGCCATTTATTCCATTAAAAAACAAAAAGCTG; Forward primer for the right homology fragment used to generate pLTPIP	This study
EF0858R-R	NNNNNNCCATGGACGCAAACGTTTGGCTGG; Reverse primer or the right homology fragment and for final overlap extension PCR to generate pLTPIP; NcoI site	This study
PIP_F3TruncA	NNNNNNTCTAGATTGATTGTTGCCTTAATGATTATTCCATCATTATATGCA; Forward primer for the left homology fragment and for final overlap extension PCR to generate pLTDPIPA; XbaI site	This study
PIP_R1TruncA	TCCTAGGCTTTCAGACAGTTGTTGCTTGCCATCTAATGTGACATTCCCTAAATCATTGGC; Reverse primer for the left homology fragment used to generate pLTDPIPA	This study
PIP_F2TruncA	GCCAATGATTTAGGGAATGTCACATTAGATGGCAAGCAACAACTGTCTGAAAGCCTAGGA; Forward primer for the right homology fragment used to generate pLTDPIPA	
PIP_R3TRuncA	NNNNNNCCATGGCCGTGCTGAAAATTGTTCCCGTTTAGAGAA; Reverse primer or the right homology fragment and for final overlap extension PCR to generate pLTDPIPA; NcoI site	This study
PIP_F1TruncB	NNNNNNTCTAGAGCCGGGATTTATTTACCTAAAGATTTTTCCAAAGAT; Forward primer for the left homology fragment and for final overlap extension PCR to generate pLTDPIPB; XbaI site	This study
PIP_R1TruncB	CGCATTCACATCAATGTTATCAACATCAGTTTTTAATCTAATTGCTTCTCTTTCTTCAACTGGTAAACGATG; Reverse primer for the left homology fragment used to generate pLTDPIPB	This study
PIP_F2TruncB	CATCGTTTACCAGTTGAAGAAAGAGAAGCAATTAGATTAAAAACTGATGTTGATAACATTGATGTGAATGCG; Forward primer for the right homology fragment used to generate pLTDPIPB	This study
PIP_R2TruncB	NNNNNNCCATGGCGCTTCAACATCTGGCATTTTCTCATTGAC; Reverse primer or the right homology fragment and for final overlap extension PCR to generate pLTDPIPB; NcoI site	This study
PIP_F1TruncC	NNNNNNTCTAGATTCAATGATATTGGCTATGATATTGATAAAAACATGGTTAGC; Forward primer for the left homology fragment and for final overlap extension PCR to generate pLTDPIPC; XbaI site	This study
PIP_R1TruncC	TTTGTTTAGTTGACCTTGAGCGTTTTGGATAACTACAATACCATCAATCGTATTCAATCTATTTATTGTGTC; Reverse primer for the left homology fragment used to generate pLTDPIPC	This study

PIP_F2TruncC	GACACAATAAATAGATTGAATACGATTGATGGTATTGTAGTTATCC AAAACGCTCAAGGTCAACTAAACAAA; Forward primer for the right homology fragment used to generate pLTDPIPC	This study
PIP_R2TruncC	NNNNNNCCATGGACACAAAGCTGTATAGAATGGCGTACTTGC; Reverse primer for the right homology fragment and for final overlap extension PCR to generate pLTDPIPC; NcoI site	This study
PIPswap-F1	NNNNNNTCTAGATTTTTGATTGTTGCCTTAATGATTATTCCATCA; Forward primer used to amplify the *E. faecalis* V583 PIP_{EF} region in pLTPdV1; XbaI site	This study
PIPswap-R1	NNNNNNCCATGGATAAAGAATTGCACCGCCAATACTAAAGAC; Reverse primer used to amplify the *E. faecalis* V583 PIP_{EF} region in pLTPdV1; NcoI site	This study
PIPcompLZ-F1	NNNNNNCTGCAGCCAGAAGCCATTGATGAAGTTAAAC; Forward primer used to clone the *E. faecalis* V583 PIP_{EF} open reading frame in pLZPIP; PstI site	This study
PIPcompLZ-R1	NNNNNNAGATCTATTGCCAAAAAGTCAAAAGCAAGAGC; Reverse primer used to clone the *E. faecalis* V583 PIP_{EF} open reading frame in pLZPIP; BglII site	This study
PIPF1-E	NNNNNNCTGCAGCCAGAAGCCATTGATGAAGTTAAACAAGTC; Forward primer for the left homology in pLZEV; PstI site	This study
PIPR1-E	AATCGTATTCAATCTATTTATTGTGTCTTGTAAATCAGTGTTACCTG TGGCTTCTTGTATTTGCGT; Reverse primer for the left homology fragment in pLZEV; BglII site	This study
PIPF2-V	ACGCAAATACAAGAAGCCACAGGTAACACTGATTTACAAGACACAA TAAATAGATTGAATACGATT; Forward primer for the right homology fragment in pLZEV	This study
PIPR2-V	NNNNNNAGATCTATTGCCAAAAAGTCAAAAGCAAGAGC; Reverse primer for the right homology fragment in pLZEV; BglII site	This study
PIPcomp28-F1	NNNNNNGGTACCCCAGAAGCCATTGATGAAGTTAAAC; Forward primer used to clone the *E. faecalis* V583 PIP_{EF} open reading frame in pPBPIP; KpnI site	This study
PIPcomp28-R1	NNNNNNGGTACCATTGCCAAAAAGTCAAAAGCAAGAGC; Reverse primer used to clone the *E. faecalis* V583 PIP_{EF} open reading frame in pPBPIP; KpnI site	This study
PIPvaruniv-F	GTCACATTAGATGGCGCYAMTMAATTG; Forward primer to amplify the PIP_{EF} variable region from sewage samples	This study
PIPvaruniv-R	CTTGAGCGTTTTGGATGGTASRAYTTAYTTTG; Reverse primer to amplify the PIP_{EF} variable region from sewage samples	This study
PIPorf-F	GCCATTGATGAAGTTAAACAAGTCGTTTCGG; Forward primer used to amplify the PIP_{EF} from *E. faecalis* mouse intestinal isolates	This study
PIPorf-R	GCTGTTCGTTTTTCCAATTTACGACGTTC; Reverse primer used to amplify the PIP_{EF} from *E. faecalis* mouse intestinal isolates	This study
VFW_2_For	TATTTTGAGTTTTATTTTGTTGTTT; Forward methyl primer for ϕVFW bisulfite sequencing	This study
VFW_2_Rev	AATCCCAATCCTATTTACCATATTC; Reverse methyl primer for ϕVFW bisulfite sequencing	This study
VPE25_1_For	GTTGGATAGTAGTAAAAATTAAAATATTTG; Forward methyl primer for ϕVPE25 bisulfite sequencing	This study
VPE25_1_Rev	ACTAACATTAATCCAAAACCAATAAAAC; Reverse methyl primer for ϕVPE25 bisulfite sequencing	This study
OG1RF_11844_For	GATATTTGTAGGTATATTTTTGTTT; Forward methyl primer for *E. faecalis* OG1RF bisulfite sequencing	This study
OG1RF_11844_Rev	AAATATTACTTTCATTAATAAAACC; Reverse methyl primer for *E. faecalis* OG1RF bisulfite sequencing	This study
mtail-F1	GACGCATTCTATGCGGACGA; Forward qPCR primer for amplifying orf_117 transcripts from ϕVPE25	This study
mtail-R1	AATGCTACACGAGGGGCAAC; Reverse qPCR primer for amplifying orf_117 transcripts from ϕVPE25	This study
PGRP-F1	TAAAGCACAGTTGGCGCAAG; Forward qPCR primer for amplifying orf_106 transcripts from ϕVPE25	This study
PGRP-R1	GCGCGTCTGGGTTTTTGTAG; Reverse qPCR primer for amplifying orf_106 transcripts from ϕVPE25	This study

capsid-F1	TGCGTGACATCGCTTACTCT; Forward qPCR primer for amplifying orf_123 transcripts from ϕVPE25	This study
capsid-R1	AGTTACCGTCTTCACGAGCA; Reverse qPCR primer for amplifying orf_123 transcripts from ϕVPE25	This study
EFrecA-F1	GATGGTGAGATGGGAGCGAG; Forward qPCR primer for amplifying *recA* transcripts from *E. faecalis* V583	This study
EFrecA-R1	CACGTCCACCAGGAGTTGTT; Reverse qPCR primer for amplifying *recA* transcripts from *E. faecalis* V583	This study
733recA-F1	CACAGCCTGACACAGGAGAA; Forward qPCR primer for amplifying *recA* transcripts from *E. faecium* 1,141,733	This study
733recA-R1	ACGTGCTTGTAACCCGACAT; Reverse qPCR primer for amplifying *recA* transcripts from *E. faecium* 1,141,733	This study

Vm^R - vancomycin resistance; Tc^R - tetracycline resistance; Em^R - erythromycin resistance; Gm^R - Gentamicin resistance; Cm^R - chloramphenicol resistant; Rf^R - rifampicin resistance; Fa^R - fusidic acid resistance; Ap^R - ampicillin resistance; Restriction sites are underlined

1. **Paulsen IT, Banerjei L, Myers GS, Nelson KE, Seshadri R, Read TD, Fouts DE, Eisen JA, Gill SR, Heidelberg JF, Tettelin H, Dodson RJ, Umayam L, Brinkac L, Beanan M, Daugherty S, DeBoy RT, Durkin S, Kolonay J, Madupu R, Nelson W, Vamathevan J, Tran B, Upton J, Hansen T, Shetty J, Khouri H, Utterback T, Radune D, Ketchum KA, Dougherty BA, Fraser CM.** 2003. Role of mobile DNA in the evolution of vancomycin-resistant *Enterococcus faecalis*. Science **299:**2071-2074.
2. **Kim EB, Kopit LM, Harris LJ, Marco ML.** 2012. Draft genome sequence of the quality control strain *Enterococcus faecalis* ATCC 29212. J Bacteriol **194:**6006-6007.
3. **Palmer KL, Carniol K, Manson JM, Heiman D, Shea T, Young S, Zeng Q, Gevers D, Feldgarden M, Birren B, Gilmore MS.** 2010. High-quality draft genome sequences of 28 *Enterococcus* sp. isolates. J Bacteriol **192:**2469-2470.
4. **Bourgogne A, Garsin DA, Qin X, Singh KV, Sillanpaa J, Yerrapragada S, Ding Y, Dugan-Rocha S, Buhay C, Shen H, Chen G, Williams G, Muzny D, Maadani A, Fox KA, Gioia J, Chen L, Shang Y, Arias CA, Nallapareddy SR, Zhao M, Prakash VP, Chowdhury S, Jiang H, Gibbs RA, Murray BE, Highlander SK, Weinstock GM.** 2008. Large scale variation in *Enterococcus faecalis* illustrated by the genome analysis of strain OG1RF. Genome Biol **9:**R110.
5. **Thurlow LR, Thomas VC, Hancock LE.** 2009. Capsular polysaccharide production in *Enterococcus faecalis* and contribution of CpsF to capsule serospecificity. J Bacteriol **191:**6203-6210.

6. **Perez-Casal J, Caparon MG, Scott JR.** 1991. Mry, a trans-acting positive regulator of the M protein gene of *Streptococcus pyogenes* with similarity to the receptor proteins of two-component regulatory systems. J Bacteriol **173:**2617-2624.
